# Treatment With Medicinal Mushroom Extract Mixture Inhibits Translation and Reprograms Metabolism in Advanced Colorectal Cancer Animal Model as Evidenced by Tandem Mass Tags Proteomics Analysis

**DOI:** 10.3389/fphar.2020.01202

**Published:** 2020-08-21

**Authors:** Boris Jakopovic, Anita Horvatić, Marko Klobučar, Andrea Gelemanović, Petra Grbčić, Nada Oršolić, Ivan Jakopovich, Sandra Kraljević Pavelić

**Affiliations:** ^1^Dr Myko San – Health from Mushrooms Co., Zagreb, Croatia; ^2^Proteomics Laboratory, Faculty of Veterinary Medicine, University of Zagreb, Zagreb, Croatia; ^3^Department of Biotechnology, University of Rijeka, Rijeka, Croatia; ^4^Mediterranean Institute for Life Sciences, Split, Croatia; ^5^Division of Animal Physiology, Faculty of Science, University of Zagreb, Zagreb, Croatia; ^6^Faculty of Health Studies, University of Rijeka, Rijeka, Croatia

**Keywords:** colorectal cancer, medicinal mushrooms, tandem mass tags proteomics, translation, cancer metabolism, unfolded protein response, multi-target, combination therapy

## Abstract

Colorectal cancer (CRC) is the third most frequent cancer type in both males and females, with about 35% of patients being diagnosed in stage IV metastatic disease. Despite advancements in treatment, life expectancy in patients with metastatic disease is still not satisfying. Due to frequent drug resistance during conventional and targeted cancer treatments, the development and testing of multi-target therapies is an important research field. Medicinal mushrooms specific isolated compounds as well as complex extract mixtures have been studied in depth, and many mushroom species have been proven to be non-toxic multi-target inhibitors of specific oncogenic pathways, as well as potent immunomodulators. In this study, we have performed a tandem mass tags qualitative and quantitative proteomic analyses of CT26.WT colon cancer tumor tissues from Balb/c mice treated with the studied medicinal mushroom extract mixture, with or without 5-fluorouracil. Besides significantly improved survival, obtained results reveal that Agarikon.1 alone, and in combination with 5-fluorouracil exert their anticancer effects by affecting several fundamental processes important in CRC progression. Bioinformatic analysis of up- and downregulated proteins revealed that ribosomal biogenesis and translation is downregulated in treatment groups, while the unfolded protein response (UPR), lipid metabolism and tricarboxylic acid cycle (TCA) are upregulated. Moreover, we found that many known clinical biomarkers and protein clusters important in CRC progression and prognosis are affected, which are a good basis for an expanded translational study of the herein presented treatment.

## Introduction

Mushrooms have been used by various civilizations for at least 7,000 years, especially in China, India, Japan, and Korea, where they have been used as a rich source of nutrients, but also as a part of traditional medicine regimens (Chang, 1999; Hobbs, 2005). Out of about 7,000 edible species, around 800 are known to possess significant pharmacological properties, and these are known as medicinal mushrooms (Boa, 2004; Barceloux, 2008; Wu et al., 2013). Modern scientific research on medicinal mushrooms started during the 1960s in Japan, and has, up till now led to the publication of more than 50,000 scientific articles on the subject. The properties of numerous mushroom species either as various extracts of whole fruiting bodies or mycelia, or isolated active substances such as polysaccharides and polysaccharide-protein complexes have been investigated. Some of the investigated isolates such as lentinan from *Lentinus edodes* (shiitake mushroom) and polysaccharopeptide Krestin (PSK) from *Trametes versicolor* (turkey tail mushroom) have been in clinical use in Japan and China since 1985 and 1977, respectively (Mizuno, 1999; Smith et al., 2002). Altogether, clinical studies of the effects of various MM preparations on humans have been published in more than 1000 papers and reports (Dai et al., 2009; Zmitrovich et al., 2019). More than 130 therapeutic effects of various mushroom species have been registered, such as antioxidant, anti-inflammatory, analgesic, antibacterial, antifungal, antiviral, cytotoxic, hepatoprotective, immunomodulatory, immunosuppressive, mitogenic, etc.) (Mizuno et al., 1995; Wasser and Weis, 1999; Hawksworth, 2001; Lindequist et al., 2005; De Silva et al., 2012; Kües and Badalyan, 2017; Sánchez, 2017; Surup et al., 2018). These effects are due to high molecular weight metabolites such as polysaccharides, proteins, lectins, and lipids, as well as a range of low molecular weight secondary metabolites such as terpenoids, lactones, alkaloids, sterols and phenolic substances (Kidd, 2000; Zhong and Xiao, 2009). Secondary metabolites are derivatives of many intermediates in primary metabolite pathways, and their abundance and variety is one of the main ecological adaptations of this group of organisms. Some of the fungal secondary metabolites such as penicillin and cephalosporins are widely used in medicine as antibiotics (Lewis, 2013).

Cancer is a complex group of diseases in which many of the molecular pathways are known to be altered in sequence and/or simultaneously. Important pathways that are involved in cancer development, and therefore also novel therapeutic targets, are nuclear factor-kappa B (NF-κB), mitogen activated protein kinase pathway (MAPK), Akt, Wnt, Notch, p53, and others. All of these pathways are known to be modulated by certain medicinal mushroom species (Zaidman et al., 2005). Also, other hallmarks of cancer can also be targeted by various medicinal mushroom compounds. Polysaccharide peptide PSP from *Trametes versicolor* reduces MDA-MB-231 breast cancer cell proliferation significantly by increasing p21WAF1/CIP1 and simultaneously decreasing cyclin D1 expression (Chow et al., 2003). Chronic inflammation is a known etiologic factor for various cancer types, including colorectal. COX-2 enzyme, which is overexpressed in various tumors and involved in prostaglandin synthesis which mediates inflammation is known to be inhibited by various mushroom species, such as *Grifola frondosa* (Zhang et al., 2002). Colorectal cancer (CRC) is one of the three most prevalent carcinomas in both males and females and the third most common cause of cancer-related deaths worldwide (Arnold et al., 2017). In 2018 alone there were 1.09 million new cases and 551,000 reported deaths from the disease (Bray et al., 2018). Most of the cases are sporadic, while 18%–35% of cases are due to hereditary predispositions, such as familial adenomatous polyposis (Lynch and de la Chapelle, 2003). Despite advancements in screening, approximately 35% of colorectal cancer patients present with stage IV metastatic disease at the time of diagnosis, and 20%–50% with stage II or III will progress to stage IV at some point during the course of their disease (Zacharakis et al., 2010). Current treatment of CRC includes surgery, radiation, chemotherapy and targeted therapy. Most commonly used chemotherapeutics in CRC treatment are 5-fluorouracil (5-FU), leucovorin, irinotecan, oxaliplatin, and capecitabine and targeted therapies such as for example monoclonal antibodies, i.e., bevacizumab (VEGF inhibitor) and cetuximab (anti-EGFR), which are sometimes used in combination with chemotherapy (Engstrom et al., 2005; Cheng et al., 2013). Although the addition of targeted therapeutics to chemotherapy has been proven effective in metastatic CRC, several clinical trials have reported failures to improve clinical outcomes in the adjuvant setting (Van Loon and Venook, 2011). Because of the pleiotropy of cancer pathways, many specific inhibitors that target one pathway have often provided modest benefit in cancer treatment. Additional major problems are therapeutic resistance and toxicity observed especially with prolonged chemotherapy. Many natural compounds such as isoflavones, curcumin, (−)-epigallocatechin-3-gallate (EGCG), resveratrol, lycopene, but also multitude of compounds as well as complex extracts from medicinal mushrooms could be classified as multi-target agents of natural origin (Zaidman et al., 2005; Sarkar et al., 2009).

*Ganoderma lucidum* and other species of medicinal mushrooms can regulate Wnt pathway which is of crucial importance in colorectal cancer. Wnt pathway induces transcriptional regulation of Axin2, c-Myc and cyclin D1 in MDA-MB-231 and 4T1 cells, and *Ganoderma lucidum* has shown a marked downregulation of Wnt3a-activated Axin2 expression (Zhang, 2017). Many of the oncogenic pathways associated with CRC such as MAPK and PI3K/AKT/mTOR converge on the translation machinery and it has been shown that global alterations in translation have an important role in cancer progression to metastasis, since apoptosis and protein synthesis is affected mainly at the level of translation (Provenzani et al., 2006). These effects are mediated by various protein complexes such as various eIFs and their negative regulators, 4E-BPs. mTOR has prominent role in protein synthesis, since functionally active mTORC1 maintains cap-dependent protein synthesis by phosphorylation and inactivation of 4E-BPs (Aras et al., 2018). In a research performed in severe combined immunodeficiency mice (SCID) injected with inflammatory breast cancer cells *Ganoderma lucidum* showed a marked reduction in expression of mTOR, p70S6K and eIF4G as well as in tumor growth (Suarez-Arroyo et al., 2013). One of the main targets of cancer therapy is the induction of apoptosis, which represents the most effective non-surgical treatment result (Pfeffer and Singh, 2018). D-fraction polysaccharide from *Grifola frondosa* induced apoptosis in 65% of hepatocellular carcinoma SMMC-7721 cell line, which is mediated by upregulation of Bax, downregulation of Bcl-2, activation of poly-(ADP-ribose)-polymerase (PARP), as well as the release of cytochrome c (Zhao et al., 2017).

Although there have been many studies in which various medicinal mushroom species have been proven to possess antitumor effects mediated by tumor signalling perturbations and/or by activation of host antitumor immunity, large scale proteomic studies of their anticancer mechanisms as well the effects on various CRC biomarkers used in the clinic have not been performed. This applies particularly to blended mushroom extracts, hypothesized to bear potentially superior biological properties in comparison to simple extracts (Shamtsyan et al., 2004; Jakopovich, 2011). Moreover, there is a lack of data addressing anticancer effects of standardized medicinal mushroom blended extracts alone or in combination with standard chemotherapy at the proteome level. We have previously determined various anticancer effects of Agarikon.1 alone and in combination with 5-fluorouracil (Durgo et al., 2013; Jakopovic et al., 2018).

Agarikon.1 effects were shown to include various immune effects such as macrophage polarization as evidenced by measuring NO and arginase, and cytokine profiles, as well as antiangiogenic properties which were established based on significant VEGF concentration reduction. Also, Agarikon.1 exhibited significant apoptotic inducing property in SW620 metastatic human colorectal adenocarcinoma cell line (Jakopovic et al., 2018). Thus, this applied syngeneic tumor model represents an advanced tumor model with a high degree of verisimilitude to the pathology of disease progression seen in human cancer patients. This model best demonstrates the interaction of tumor cells with tumor microenvironment cells that can facilitate or prevent tumor progression. In this follow-up research, we have identified up- and downregulated proteins and protein clusters relevant in colorectal cancer progression by using high-resolution qualitative and quantitative proteomic analysis in combination with comprehensive bioinformatics analyses. Some of them are used as biomarkers in the clinic and have been analyzed herein, to discern the most significant antitumor processes induced by the tested substances in the presented model of late-stage colorectal cancer.

## Materials and Methods

### Animals

Male Balb/c mice, approximately 2 months old, weighing 20-25 grams were obtained from the Rudjer Boskovic Institute, Facility for laboratory animals, Zagreb. Animals were kept under conventional housing conditions and were maintained on a pellet diet (Standard Diet 4RF 21 GLP certificate, Mucedola, Italy), given water *ad libitum*, and subjected to an equal 12-h light/dark cycle in accordance with institutional guidelines. Experimental groups comprised 10 mice each. Animal studies were approved by the University’s of Zagreb, Department of Biology Ethics Committee (approval code: 251-58-10617-16-14) and performed in compliance with the guidelines in force in the Republic of Croatia (the Croatian Animal Welfare Law (NN, 135/2006 and 37/2013)) and according to the European Directive 2010/63/EU.

### Tumor Cells

CT26.WT (ATCC^®^ CRL-2638™) was obtained from American Type Culture Collection (ATCC). This is an N-nitroso-N-methylurethane-(NNMU) induced murine colorectal cell line syngeneic with Balb/c mice. This cell line shares molecular features with sporadic, aggressive, undifferentiated (stage IV), therapy-refractory human colorectal carcinoma cells, it is easily implanted and metastasizes readily (Castle et al., 2014). The cells were propagated, screened for mycoplasma contamination (MycoAlert™ PLUS mycoplasma detection kit, Lonza Walkersville, Walkersville, MD) and subcultured in accordance to the distributor’s protocol. Cells were grown in RPMI-1640 medium with 10% FBS, penicillin (100 U/ml) and streptomycin (100 μg/ml) and maintained at 37°C with 5% CO_2_ in a humidified atmosphere. After harvesting and preparation of cells, their total number and viability were determined by counting in a Neubauer chamber using Trypan Blue Dye, and was always found to be at least 95% viable.

### Tested Substances

Medicinal mushroom extract mixture Agarikon.1 (LOT:1100517) was provided by Dr Myko San – Health from Mushrooms Co, Croatia. This preparation has been registered by the Ministry of Health and Social Welfare of the Republic of Croatia as a dietary supplement (registration number MZ 0813411210) (Jakopovich, 2011). It is produced from a hot water extract which is precipitated with ethanol and subsequently freeze-dried. This tablet preparation contains a mixture of *Lentinus edodes*, *Ganoderma lucidum*, *Agaricus brasiliensis* (=*blazei* ss. Heinem.), *Grifola frondosa*, *Pleurotus ostreatus*, and *Trametes versicolor* medicinal mushroom species, in equal amounts i.e. 125 mg each per tablet. One 1000 mg tablet therefore contains 750 mg of mushroom polysaccharides per tablet, combined with excipients such as inulin, talc, magnesium stearate, and silica. Its antitumor and antioxidative effects on certain tumor lines have been established (Durgo et al., 2013; Jakopovic et al., 2018).

5-fluorouracil (manufacturer: Sandoz), the antimetabolite class chemotherapy drug was supplied at a concentration of 50 mg/ml in sterile aqueous solution, pH 8.6 to 9.0, and stored at 4°C in aluminum covered containers. Immediately prior to use, it was diluted in sterile distilled water.

### Experimental Design and Procedures

**Figure 1:**
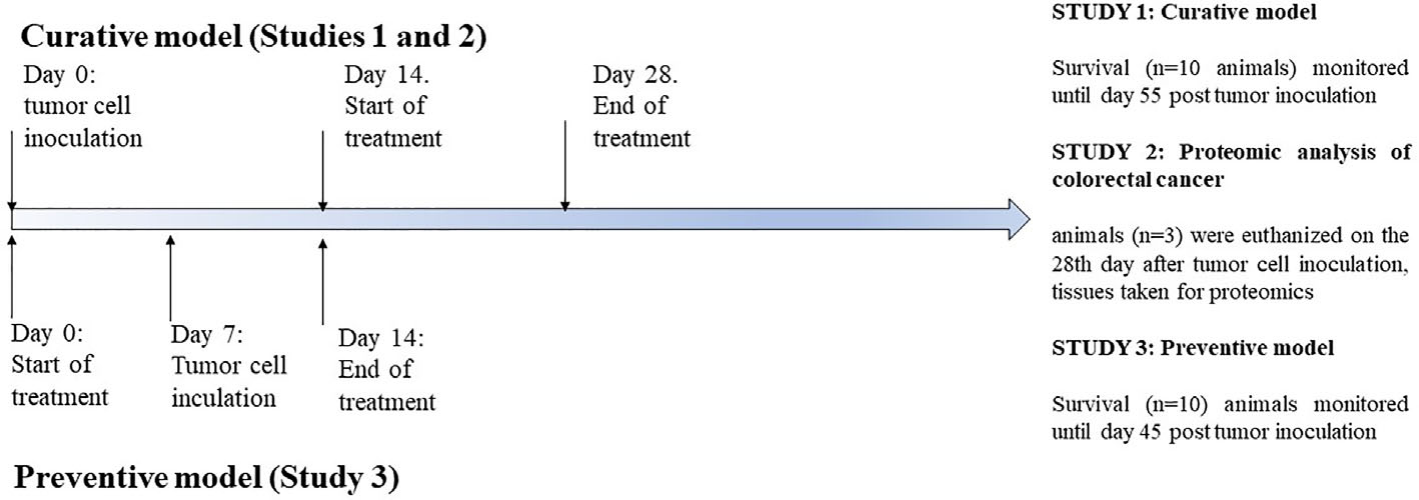
Treatment design for the studies 1, 2, and 3.

Prior to treatment animals were divided into three studies (**Figure 1**). The mice in study 1 were injected subcutaneously in the right flank with 1 x 10^6^ viable CT26.WT cells in 100 μl of sterile PBS. Treatment of animals with tumors was started when the tumor was developed in 100% of the animals with a palpable solid tumor mass (≥ 700 mm^3^, 14 days post-implantation), which is an advanced stage of the tumor. Mice were randomly divided into 4 groups (n=10 mice/group) and treated with 1,200 mg/kg of Agarikon.1 by oral gavage during 14 days continuously, or with 5-FU intraperitoneally (30 mg/kg on days 1.–4. and 15 mg/kg on 6., 8., 10., and 12. day of treatment), or with both Agarikon.1 and 5-FU in aforementioned concentrations. The control was given the same volume of saline by oral gavage. 5-FU was administered metronomically, and for both preparations the doses were calculated by interspecies allometric scaling (Nair and Jacob, 2016). The basis for the dose calculation of Agarikon.1 was the recommended daily dose of this dietary supplement used by patients. The survival was monitored until day 55. after tumor inoculation, after which the remaining animals were euthanized. In the second study, mice were treated in the same manner as the first, but the animals (n=3 per group) were euthanized on the 28th day after tumor cell inoculation, after which the tumor tissues were collected and stored in liquid nitrogen immediately until proteomic analysis. Third study comprised only one group (n=10 mice/group) which was treated with Agarikon.1 (1200 mg/kg) intragastrically for one week before and one week after tumor cell inoculation (1 x 10^6^ CT26.WT cells). The survival in this group was monitored until day 45. after tumor cell inoculation, after which the remaining animals were euthanized. In this manner, mice in both curative (first study) and preventive (third study) groups received treatment during equal time period. In order to analyze the inhibitory effects of tested substances on tumor growth, tumor length (L) and width (W) was measured and tumor volume (mm^3^) was calculated as [V=(L x W^2^)/2]. Data was analysed by Kruskal-Wallis ANOVA. Further analysis of the differences between the groups was made with multiple comparisons of mean ranks for all groups. Statistical analyses were performed using STATISTICA 12 software (StatSoft, Tulsa, OK, USA). The data was considered significant at *p* < 0.05.

### Survival Analysis

Animal life span was evaluated by daily surveillance of spontaneous death or by selective euthanasia of animals showing signs of pain and suffering according to established criteria. Kaplan-Meier statistical analysis (log rank statistics) as well as overall survival were utilized to compare survival. Analyses were performed using MedCalc (Version 19.1.3) and the significance level was 5% (*p* < 0.05).

### Preparation of Tissue Homogenates

Tumor tissue was mechanically grinded and homogenized in a mortar with liquid nitrogen. Homogenized tissue was dissolved in 1 ml of a lysis buffer [7 M urea/2 M thiourea (Sigma-Aldrich, USA), 4% (w/v) CHAPS (Sigma-Aldrich), 1% (w/v) dithiothreitol (DTT) (Sigma-Aldrich)], and 1× protease inhibitor cocktail (Roche, Switzerland). The obtained lysate was subjected to sonication with 4 mm probe, power of 6 W (MicrosonTM, PGC Scientifics, USA), four times for 10 s. After sonication, the samples were incubated for 1 h at room temperature with gentle agitation on the thermo shaker (Eppendorf, Germany). After that the samples were centrifuged for 45 min at 14,000 rpm and 4°C (Eppendorf, Germany). The supernatant was collected, aliquoted and stored at −80°C for further analyses. Protein concentrations were determined using the Qubit™ fluorometric (Invitrogen,USA) quantitation platform. Before measurement of protein concentration the platform was calibrated with Qubit protein standards (Invitrogen,USA).

### Preparation of Samples and Mass Spectrometry Analysis

From each sample, acetone-precipitated proteins were dissolved in triethyl ammonium bicarbonate (TEAB, Thermo Scientific, Rockford, USA) and subjected to reduction, alkylation, digestion and labelled using 10-plex Tandem Mass Tag reagents according to manufacturer instructions (Thermo Scientific, Rockford, USA). In short, 35 μg of total plasma proteins from samples and internal standard (pool of all samples) were dissolved in 50 μl 0.1M TEAB, reduced by adding 2.5 μl of 200 mM DTT (60 min, 55°C) (Sigma Aldrich, St. Louis, MO, USA), alkylated by adding 2.5 μl of 375mM IAA (30 min, room temperature in the dark) (Sigma Aldrich, St. Lois, MO, USA) and acetone-precipitated (addition of 300 μl, overnight, −20°C). Protein pellets were collected subsequently by centrifugation (8,000×g, 4°C), dissolved in 50 μl 0.1M TEAB and digested using 1 μl of trypsin (1 mg/ml, Promega; trypsin-to-protein ratio 1:35, at 37°C overnight).

TMT label reagents were equilibrated to room temperature, resuspended in anhydrous acetonitrile LC-MS grade (Thermo Scientific, Rockford, USA) and added to each sample. Labelling was performed for 1 hour at room temperature and then quenched by adding 5% hydroxylamine (Thermo Scientific, Rockford, USA) for 15 min. Samples were then combined at equal amounts and 5 μg of each mixed sample set was vacuum-dried and stored at - 80°C for LC-MS/MS analysis. High resolution LC-MS/MS analysis of TMT-labelled peptides was carried out using an Ultimate 3000 RSLCnano system (Dionex, Germering, Germany) coupled to a Q Exactive Plus mass spectrometer (Thermo Fisher Scientific, Bremen, Germany) as described elsewhere (Horvatić et al., 2019). In short, labelled peptides were desalted on the trap column for 12 min at the flow rate of 15 μl/min and separated on analytical column (PepMap™ RSLC C18, 50 cm×75 μm) using a linear gradient of 5%–45% mobile phase B over 120 min at the flow rate of 300 nl/min. Mobile phase A consisted of 0.1% formic acid in water, while B contained 0.1% formic acid in 80% ACN. Ionisation was achieved using nanospray Flex ion source (Thermo Fisher Scientific, Bremen, Germany) equipped with a 10 μm-inner diameter SilicaTip emitter (New Objective, USA). The MS operated in positive ion mode using DDA Top8 method. Full scan MS spectra were acquired in range from m/z 350.0 to m/z 1800.0 with a resolution of 70,000, 120 ms injection time, AGC target 1×10^6^, a ± 2.0 Da isolation window and the dynamic exclusion 30 s. HCD fragmentation was performed at step collision energy (29% and 35% NCE) with a resolution of 17,500 and AGC target of 2×10^5^. Precursor ions with unassigned charge state, as well as charge states of +1 and more than +7 were excluded from further fragmentation.

### Protein Identification and Quantification

For peptide identification and relative quantification the SEQUEST algorithm implemented into Proteome Discoverer (version 2.3, Thermo Fisher Scientific) was used. Database search against *Mus musculus* FASTA files downloaded from NCBI database (11/4/2019, 185475 entries) was performed according to the following parameters: two trypsin missed cleavage sites, precursor and fragment mass tolerances of 10 ppm and 0.02 Da, respectively; carbamidomethyl (C) fixed peptide modification, oxidation (M), and TMT sixplex (K, peptide N-terminus) dynamic modifications. The false discovery rate (FDR) for peptide identification was calculated using the Percolator algorithm within the Proteome Discoverer workflow and was set at 1% FDR. Only proteins with at least two unique peptides and 5% FDR were reported as identified. Protein quantification was accomplished by correlating the relative intensities of reporter ions extracted from tandem mass spectra to that of the peptides selected for MS/MS fragmentation. The internal standard was used to compare relative quantification results for each protein between the experiments. The mass spectrometry proteomics data have been deposited to the ProteomeXchange Consortium ***via*** the PRIDE partner repository with the dataset identifier PXD018827.

### Western Blot Analysis

For the Western blot analysis a total of 50 μg proteins isolated from tumor tissues obtained from control and treated groups of animals were resolved on 12% SDS polyacrylamide gels using the Mini-PROTEAN Tetra cell (BioRad, USA). The PVDF membranes (BioRad, USA) were incubated with primary antibodies raised against Rps3 (1:500; rabbit pAb; St John’s Laboratory,UK) and Apoa2 (1:500; rabbit pAb; St John’s Laboratory,UK) at 4°C overnight. Secondary antibody linked to anti-rabbit (1:2000, Cell Signaling Tehnology, USA) was used. The signal was visualized by Amersham ECL Western Blotting Detection Reagent (GE Healthcare, USA) on the ImageQuant LAS 500 (GE Healthcare, USA) and α-tubulin (1:1,000, mouse mAb, SigmaAldrich, USA) was used as a loading control. The signal intensities of particular bands were normalized with the intensity of the loading control and compared in Quantity One software (Bio-Rad, USA). The values are expressed as the average ± SEM.

### Statistical Analysis

For comparison of protein abundances data was normalized by using internal standard. After exclusion of outliers using Dixon test and proteins with less than two unique peptides, Kruskal-Wallis test was performed to test the difference in protein abundance between the groups. For proteins that were significantly different, Conover post-hoc test was performed for pairwise multiple comparisons. In all cases, values of *p* < 0.05 were considered significant. Fold changes (FC) have been calculated as follows: FC = log2[mean(Treatment)/mean(Control)]. Statistics were performed using Rstudio (v3.2.2). Differences in relative expression status of proteins obtained by Western blot analysis were analysed by ANOVA in Microsoft Excel. Statistical significance was set at *p* < 0.05 were considered statistically significant.

### Bioinformatic Analysis

Proteins GI accession numbers were converted into official gene symbol by DAVID conversion tool (https://david.ncifcrf.gov/conversion.jsp). If any protein GI accession number was not identified, it was run through SmartBLAST to identify highly similar proteins (https://blast.ncbi.nlm.nih.gov/smartblast/). In order to assign biological functions, identified proteins were further subjected to UniProt (http://uniprot.org/) and PANTHER (http://pantherdb.org) database searches. Enrichment analysis of protein-protein interaction networks was performed using STRING database v11.0 (https://string-db.org/), with the selection of appropriate organism and default settings with the exception of no more than 5 interactors to show in 1st shell and the minimum required interaction score set to high (0.700). Pathway analyses for up- or down-accumulated clusters of proteins were performed for each treatment group separately using REACTOME software (http://www.reactome.org/) and STRING database. The REACTOME performs an enrichment test to determine whether any REACTOME pathways are enriched in the submitted data. A binomial test was used to calculate the probability. The *p*-values are corrected for multiple testing (Benjamini-Hochberg procedure) that arises from evaluating the submitted list of identifiers against every pathway. The pathway with the corrected *p*-value less than 0.05 was considered to be significantly enriched

## Results

### Survival and Tumor Volume Analysis

**Figure 2:**
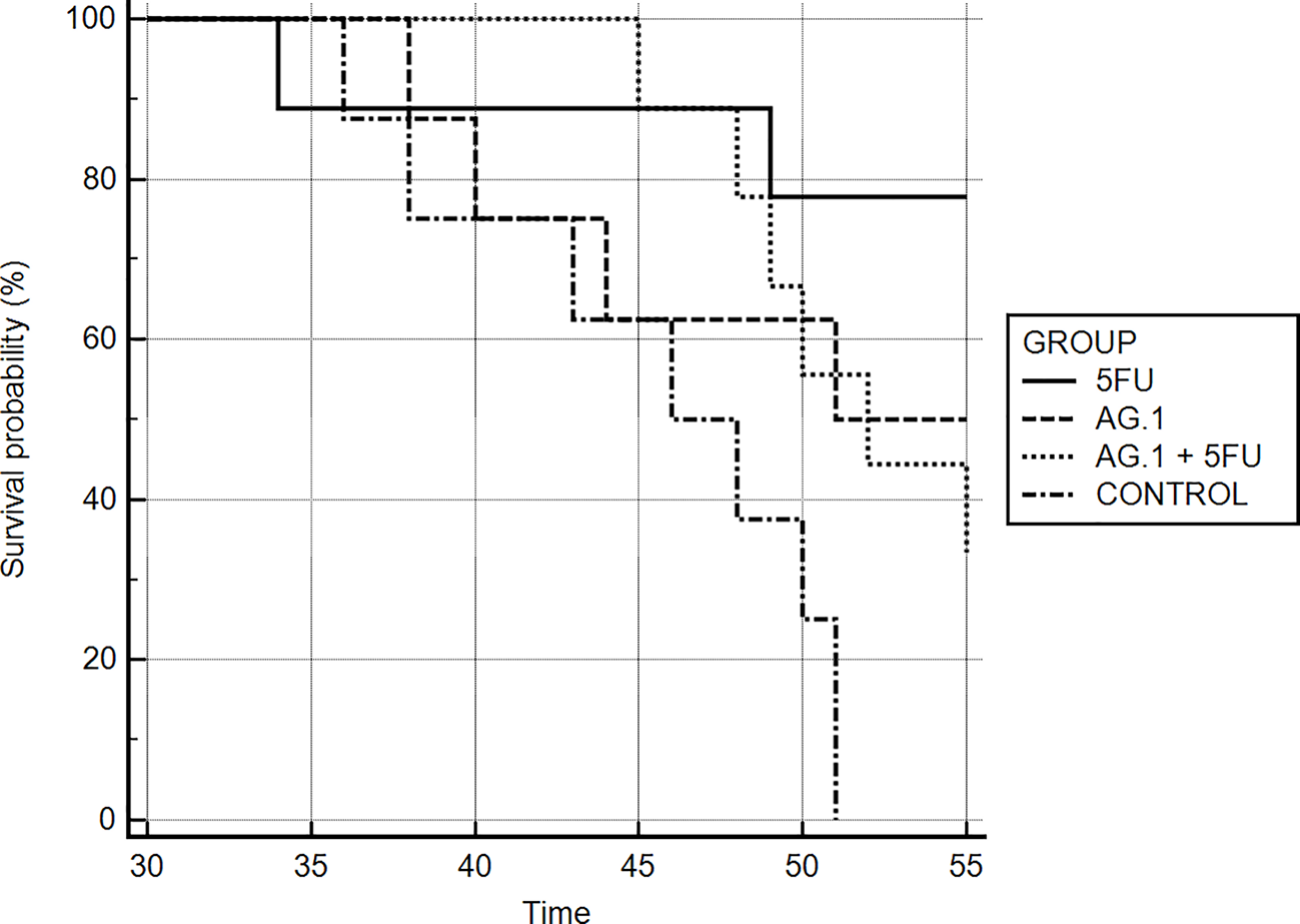
Kaplan-Meier survival curves of mice bearing CT26.WT tumors in different treatment groups.

**Figure 3:**
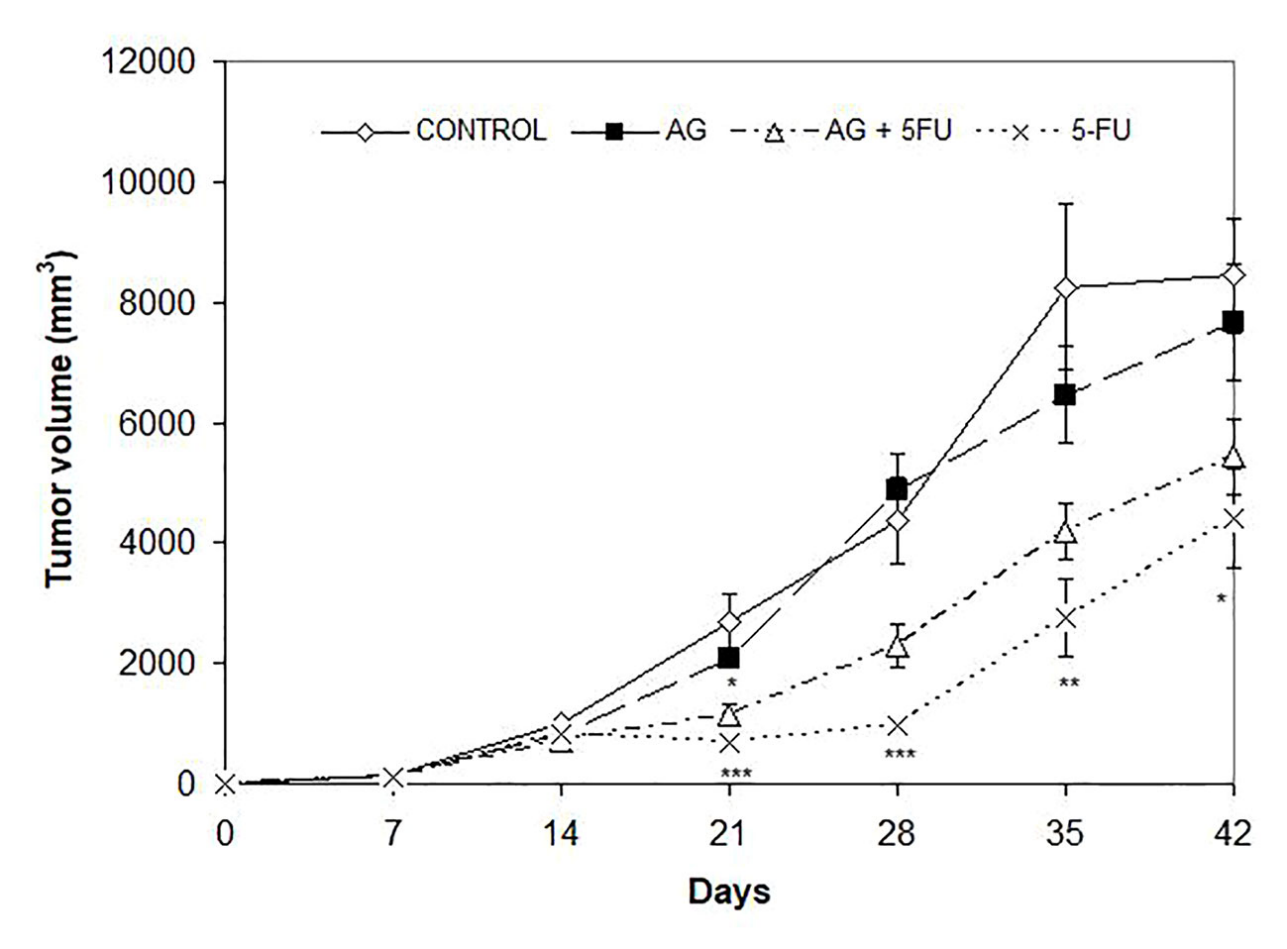
Effect of Agarikon.1 (AG), 5-fluorouracil (5-FU), and their combination on tumor volume. Treatment started 14 days after s.c. inoculation of CT26.WT (1×10^6^/mouse) cells with 1200 mg/kg of Agarikon.1 by oral gavage during 14 days continuously, or with 5-FU intraperitoneally at dose of 30 mg/kg on days 1.-4. and 15 mg/kg on 6., 8., 10., and 12. day of treatment. Volumes were determined once weekly for six weeks. *p <; 0.05; **p < 0.01; ***p < 0,001, versus control group. Bars show means ± SEM.

Mice bearing CT26.WT tumors administered either with Agarikon.1 (AG.1), 5-fluorouracil (5-FU) or AG.1 in combination with 5-FU two weeks after tumor inoculation showed a significant increase in life span with respect to control. The Kaplan-Meier (log-rank) survival analysis curve was plotted for the curative treatment subsection (**Figure 2**), and the statistical relevance for the same subsection along with overall survival rate is indicated in **Table 1**. In the preventive subsection, all animals treated with AG.1 were still alive (n=10) on day 45. after tumor cell inoculation. The length of survival observation in this subsection was based according to literature, where the usual life span of 34 ± 6.2 days is observed in animals which are not treated (Ojo-Amaize et al., 2007; Ito et al., 2015). Although the tumor volume reduction was observed in all treatment groups, statistical significance in comparison to control was only noted in a group treated with 5-fluorouracil alone (**Figure 3**).

**Table 1:** Survival analysis of Balb/c mice bearing CT26.WT tumor after treatment.

Experimental groups	Overall survival rates	Kaplan-Meier analysis	
Curative Subsection	% of survivors on day 55. post tumor inoculation	*p*	*χ^2^*
AG.1	50	0.0650	3.404
5-FU	77.7	0.0028	8.948
AG.1 + 5-FU	33.3	0.0189	5.513
Preventive subsection	% of survivors on day 45. post tumor inoculation		
AG.1	100	NA	NA

AG.1, Agarikon.1; 5-FU, 5-fluorouracil; NA, not applicable, survival of control group based on literature.

### Proteomics

After exclusion of isomers and unnamed protein products, tandem mass tag proteomic analysis of tumor tissue revealed a total of 95 up- or downregulated proteins between the treatment groups and control mice, out of which 36 were up- and 59 were downregulated (**Tables 2****–****4**).

**Table 2:** List of differentially regulated proteins in AG.1 treated group *vs* control group. Positive fold-change value denotes up-accumulation and negative fold change value denotes down-accumulation in comparison with control.

Gene symbol	Protein name	*P*-value (FDR)	Fold change (log2 vs control)	Biological process
Apoa2	Apolipoprotein A-II	0.004158	1.621488	Lipid transport and metabolism, retinoid transport, chylomicron remodeling
Acot7	Cytosolic acyl coenzyme A thioester hydrolase	0.028981	1.272644	Fatty acid metabolism, lipid metabolism
Myl6	Myosin light polypeptide 6	0.031223	1.258035	Muscle contraction, GTPase signaling
Dnajc3	DnaJ homolog subfamily C member 3	0.013975	1.018262	Stress response, translation regulation, unfolded protein response
Cdk11b	Cyclin-dependent kinase 11B	0.013975	1.018262	Cell cycle
Fh	Fumarate hydratase, mitochondrial	0.034637	0.656297	DNA damage, DNA repair, tricarboxylic acid cycle
Cavin2	Caveolae-associated protein 2	0.021712	0.396121	Transcription regulation, lipid metabolism
Rpsa	40S ribosomal protein SA	0.010806	-1.2591273	Translation, cell-cell adhesion
Anxa7	Annexin A7	0.027257	-0.7312487	Cell signaling, cell population proliferation, integrin binding, ECM interactions
Sbds	Ribosome maturation protein SBDS	0.03173	-0.6586195	Ribosome biogenesis and assembly
Rps3	40S ribosomal protein S3	0.005865	-0.4711172	Transcription, transcription regulation, translation regulation, cell cycle

**Table 3:** List of differentially regulated proteins in 5-FU treated group *vs* control group. Positive fold-change value denotes up-accumulation and negative fold change value denotes down-accumulation in comparison with control.

Gene symbol	Protein name	*P*-value (FDR)	Fold change (log2 vs control)	Biological process
Apoa2	Apolipoprotein A-II	0.004158	0.771235	Lipid transport and metabolism, retinoid transport, chylomicron remodeling
Mt-1	Metallothionein-1	0.012015	0.674153	Cellular metal ion homeostasis, nitric oxide mediated signal transduction
Ptma	Prothymosin alpha	0.016023	0.518607	Histone exchange, negative regulation of apoptotic process, cell cycle
Ces1c	Carboxylesterase 1C	0.023659	0.4521	Lipid catabolic process, response to metal ions
Rpl29	60S ribosomal protein L29	0.006359	0.429547	Translation
Cavin2	Caveolae-associated protein 2	0.021712	0.404603	Transcription regulation, lipid metabolism
Cdk11b	Cyclin-dependent kinase 11B	0.013975	0.377805	Cell cycle
Dnajc3	DnaJ homolog subfamily C member 3	0.013975	0.377805	Stress response, translation regulation, unfolded protein response
Ipo7	Importin-7	0.048261	0.316322	Innate immune response, protein import into nucleus
S100a9	Protein S100-A9	0.037099	-1.70807	Apoptosis, autophagy, chemotaxis, immunity, inflammatory response, innate immunity
Anxa5	Annexin A5	0.02236	-0.74139	Calcium ion transmembrane transport, apoptosis

**Table 4:** List of differentially regulated proteins in AG.1 and 5-FU treated group *vs* control group. Positive fold-change value denotes up-accumulation and negative fold change value denotes down-accumulation in comparison with control.

Gene symbol	Protein name	*P*-value (FDR)	Fold change (log2 vs control)	Biological process
Myh4	Myosin-4	0.017948	2.160907	Muscle contraction
Apoa2	Apolipoprotein A-II	0.004158	2.160647	Lipid transport and metabolism, retinoid transport, chylomicron remodeling
Myl6	Myosin light polypeptide 6	0.031223	1.709041	Muscle contraction, GTPase signaling
Ces1c	Carboxylesterase 1C	0.023659	1.657592	Lipid catabolic process, response to metal ions, interferon signaling
Myh1	Myosin, heavy polypeptide 1, skeletal muscle, adult	0.018684	1.657005	Actin filament binding
Myl1	Myosin light chain 1/3, skeletal muscle isoform	0.039277	1.605843	Muscle contraction
Myh2	MCG140437, isoform CRA_d	0.012003	1.573102	Actin mediated cell contraction, immune system
Acot7	Cytosolic acyl coenzyme A thioester hydrolase	0.028981	1.535928	Fatty acid metabolism, lipid metabolism
Etfb	Electron transfer flavoprotein subunit beta	0.045959	1.515519	Fatty acid beta-oxidation, electron transport chain, ECM organization
Ces1d	Carboxylesterase 1D	0.018917	1.27988	Lipid degradation, lipid metabolism
Ces1b	Carboxylic ester hydrolase	0.031849	1.249791	Lipid catabolic process, response to metal ions, interferon signaling
Ces1	Liver carboxylesterase 1	0.031849	1.249791	Lipid catabolic process, response to metal ions, interferon signaling
Fh	Fumarate hydratase, mitochondrial	0.034637	1.236643	DNA damage, DNA repair, tricarboxylic acid cycle
Apoa4	Apolipoprotein A-IV	0.020958	1.149576	Lipid transport and metabolism, retinoid transport, chylomicron remodeling, immune response
Dnajc3	DnaJ homolog subfamily C member 3	0.013975	1.110521	Stress response, translation regulation, unfolded protein response
Cdk11b	Cyclin-dependent kinase 11B	0.013975	1.110521	Cell cycle
Serpina1c	Alpha-1-antitrypsin 1-3	0.036681	1.0573	Negative regulation of endopeptidase activity
Eno3	Beta-enolase	0.029494	1.032035	Glycolytic process, response to drug, hypoxia tolerance
Serpina1a	Alpha-1-antitrypsin 1-1	0.029013	0.986873	Acute phase response, negative regulation of endopeptidase activity
N/A	Ig gamma-3 chain C region	0.042381	0.516351	Immunoglobulin receptor binding
Syncrip	Heterogeneous nuclear ribonucleoprotein Q	0.02107	-1.8252	mRNA processing, mRNA splicing, translation
Ptbp3	Polypyrimidine tract-binding protein 3	0.04308	-1.5654	Differentiation, mRNA processing, mRNA splicing
Sh3bgrl3	SH3 domain-binding glutamic acid-rich-like protein 3	0.02477	-1.3628	Electron transport chain, cell redox homeostasis
Cdc42	Cell division control protein 42 homolog	0.03274	-1.2917	Actin cytoskeleton organization, cell cell adhesion, regulation of cell growth and division, DNA replication, angiogenesis
Rpsa	40S ribosomal protein SA	0.01081	-1.2857	Translation, cell-cell adhesion
Hnrnpk	Heterogeneous nuclear ribonucleoprotein K	0.01024	-1.269	mRNA processing, mRNA splicing, transcription, transcription regulation
Cavin1	Caveolae-associated protein 1	0.04802	-1.251	Transcription, transcription regulation, transcription termination
Ddx3x	ATP-dependent RNA helicase DDX3X	0.02665	-1.2316	Apoptosis, chromosome partition, immunity, innate immunity, ribosome biogenesis, transcription and translation regulation
Hnrnpd	Heterogeneous nuclear ribonucleoprotein D0	0.00449	-1.1753	Biological rhythms, transcription regulation
Rpl36a	60S ribosomal protein L36a	0.013	-1.1079	Translation
Rpl36a-ps1	MCG113838	0.013	-1.1079	Translation
Hmgb1	High mobility group protein B1	0.00315	-0.9337	Adaptive immunity, autophagy, chemotaxis, DNA damage, DNA recombination, DNA repair, immunity, inflammatory response, innate immunity
Capg	Macrophage-capping protein	0.01695	-0.9243	Actin filament capping, cell cycle
Rpl24	60S ribosomal protein L24	0.00842	-0.9223	Translation
Ppia	Peptidyl-prolyl cis-trans isomerase A	0.00659	-0.9137	Regulation of viral genome replication, protein refolding, positive regulation of protein secretion, lipid droplet organization
Hnrnpf	Heterogeneous nuclear ribonucleoprotein F	0.02066	-0.9057	mRNA processing, mRNA splicing
Rbmx	RNA-binding motif protein, X chromosome	0.02066	-0.9057	mRNA processing, mRNA splicing, Transcription
Tcp1	T-complex protein 1 subunit alpha	0.03015	-0.8699	Regulation of macrophage apoptotic process, positive regulation of telomerase activity, toxin transport, protein stabilization
Aars	Aars protein	0.04044	-0.8244	Alanyl-tRNA aminoacylation, translation, integrin cell surface interactions
Anxa7	Annexin A7	0.02726	-0.8209	Cell signaling, cell population proliferation, integrin binding, ECM interactions
Prdx2	Peroxiredoxin-2	0.04967	-0.8049	Redox homeostasis, cellular response to oxidative stress, homeostasis of number of cells, apoptosis, immunity
Myc	Myc proto-oncogene protein	0.01002	-0.7689	Transcription, transcription regulation
Hnrnpu	Heterogeneous nuclear ribonucleoprotein U	0.04623	-0.7688	Biological rhythms, cell cycle, cell division, differentiation, mitosis, mRNA processing, mRNA splicing, transcription, transcription regulation
Rps21	40S ribosomal protein S21	0.02436	-0.7589	Translation
Arf1	ADP-ribosylation factor 1	0.0149	-0.7558	ER-Golgi transport, protein transport, actin filament organization
Pcna	Proliferating cell nuclear antigen	0.02122	-0.7097	DNA damage, DNA repair, DNA replication
Nolc1	Nucleolar and coiled-body phosphoprotein 1	0.01141	-0.6908	Positive regulation of cell population proliferation, positive regulation of transcription, DNA-templated, regulation of translation
Eif3j2	Eukaryotic translation initiation factor 3 subunit J-B	0.04899	-0.6729	Protein biosynthesis, formation of cytoplasmic translation initiation complex
Rpl13	60S ribosomal protein L13	0.03669	-0.665	Translation
Rps3	40S ribosomal protein S3	0.00587	-0.6491	Apoptosis, cell cycle, cell division, DNA damage, DNA repair, mitosis, transcription, transcription regulation, translation regulation
Sbds	Ribosome maturation protein SBDS	0.03173	-0.6382	Ribosome biogenesis
Arhgdia	Rho GDP-dissociation inhibitor 1	0.0138	-0.6057	Rho protein signal transduction, regulation of actin cytoskeleton reorganization
Hspa9	Stress-70 protein, mitochondrial	0.03181	-0.6036	Chaperone cofactor-dependent protein refolding, cellular response to unfolded protein, negative regulation of cell death
Sar1a	GTP-binding protein SAR1a	0.00524	-0.5923	ER-Golgi transport, protein transport, transport
Hmgb2	High mobility group protein B2	0.00288	-0.5881	Chemotaxis, DNA recombination, immunity, inflammatory response,innate immunity, transcription regulation
Myh10	Myosin-10	0.04511	-0.5574	Cell adhesion, cell shape
Ssb	Sjogren syndrome antigen B, isoform CRA_a	0.01179	-0.5558	RNA processing, RNA binding
Eif4a1	Eukaryotic initiation factor 4A-I	0.02086	-0.549	Cytoplasmic translational initiation
Eif4a2	Eukaryotic initiation factor 4A-II	0.04259	-0.5107	Protein biosynthesis, cytoplasmic translational initiation
Elavl1	ELAV-like protein 1	0.04627	-0.4759	RNA-binding, regulation of stem cell population maintenance, positive regulation of translation
Cap1	Adenylyl cyclase-associated protein 1	0.0129	-0.4536	Actin polymerization or depolymerization, ameboidal-type cell migration, actin cytoskeleton organization
Ywhae	14-3-3 protein epsilon	0.02834	-0.45	MAPK cascade, positive regulation of protein export from nucleus, negative regulation of calcium ion export across plasma membrane
Gm10260	Predicted gene 10260	0.03753	-0.4143	Translation, structural constituent of ribosome
Rps18-ps5	MCG116671	0.03753	-0.4143	Translation, structural constituent of ribosome
Rps18	40S ribosomal protein S18	0.03753	-0.4143	Translation
Usp14	Ubiquitin carboxyl-terminal hydrolase 14	0.04611	-0.414	Immunity, Innate immunity, Ubl conjugation pathway
Ddx39b	Spliceosome RNA helicase Ddx39b	0.02855	-0.4137	Transcription, translation, peroxisomal lipid metabolism, fatty acid metabolism
Rps13	40S ribosomal protein S13	0.0138	-0.3902	Negative regulation of RNA splicing, translation
Rplp0	60S acidic ribosomal protein P0	0.00946	-0.3633	Cytoplasmic translation, ribosomal large subunit assembly
Prdx6	Peroxiredoxin-6	0.01493	-0.3017	Lipid degradation, lipid metabolism, response to oxidative stress

These proteins have various molecular and biological functions, determined by the Uniprot database search, as well as by use of PANTHER GO analysis (**Figure S1**). In the AG.1 group (**Table 2**, **Figures S1A, B**), differential proteins in abundance (upregulated) are classified into six categories according to molecular function: binding, catalytic activity, molecular function regulator, molecular transducer activity, structural molecule activity, and transporter activity. These are involved in various biological processes, such as biological regulation, cellular process, localization, metabolic process (i.e., apolipoprotein A-II, fumarate hidratase), and multicellular organismal process. Downregulated proteins in this group are involved in three molecular functions: binding, catalytic activity, and structural molecule activity and are implicated in the following biological processes: biological regulation, cellular component organization and biogenesis (i.e., 40S ribosomal protein SA, ribosome maturation protein SBDS), cellular process, localization and metabolic process. Upregulated proteins in group treated with 5-FU showed that the differential proteins are classified into six categories according to molecular function (binding, catalytic activity, molecular function regulator, molecular transducer activity, structural molecule activity) and biological process [biological regulation, cellular process, localization, metabolic process (i.e., 60S ribosomal protein L29, carboxylesterase 1C), multicellular organismal process, response to stimulus]. Downregulated proteins in the same group are assigned one molecular function (binding) and two biological processes [cellular process, metabolic process (i.e., protein S100-A9)] (**Table 3**, **Figures S1C, D**). Finally, the upregulated proteins in combinatorial group (**Table 4**, **Figures S1E, F**) (treated with both AG.1 and 5-FU) are classified into six categories with regards to molecular function (binding, catalytic activity, molecular function regulator, molecular transducer activity, structural molecule activity) and biological processes [biological regulation, cellular process, localization, metabolic process (i.e., beta enolase, fumarate hydratase), multicellular organismal process, response to stimulus] while downregulated proteins of the same groups possess the following molecular functions (binding, catalytic activity, molecular function regulator, molecular transducer activity, structural molecule activity) and are involved in the following biological processes: biological regulation, cellular component organization and biogenesis (i.e., 40S ribosomal protein S21), cellular process (i.e., Myc proto-oncogene protein, cell division control protein 42 homolog), immune system process (i.e., high mobility group protein B1), localization, metabolic process, multicellular organismal process, response to stimulus.

**Figure 4:**
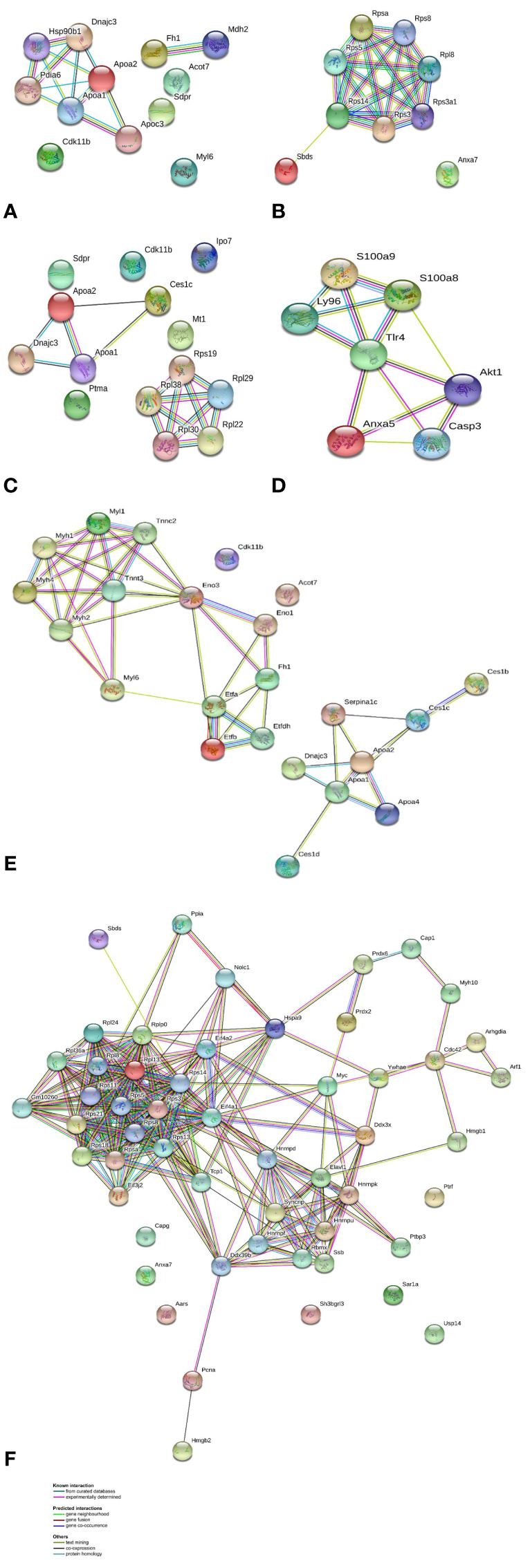
Protein-protein interaction networks identified using STRING for differential proteins in abundance. Different line colors represent the types of evidence for association. Gene names are shown. **(A)** AG.1 group up-accumulated proteins, **(B)** AG.1 group down-accumulated proteins, **(C)** 5-FU group up-accumulated proteins, **(D)** 5-FU group down-accumulated proteins, **(E)** combinatorial group (AG.1 with 5-FU) up-accumulated proteins, and **(F)** combinatorial group (AG.1 with 5-FU) down-accumulated proteins.

In order to explore the likely mechanisms in more detail, protein-protein interaction network analysis was performed on the differentially regulated proteins using STRING, in accordance with previously set parameters. For each group, the up- and downregulated proteins were mapped to a different interaction network (**Figure 4**).

For the group treated with AG.1, the two main sub-networks include 6 proteins (Dnajc3, Hsp90b1, Pdia6, Apoa1, Apoa2, Apoc3), which are mainly involved in unfolded protein response (UPR) and lipid metabolism and a sub-network of 2 proteins (Fh1, Mdh2) mainly involved in pyruvate metabolism and citric acid cycle, while the main cluster of proteins which are down-regulated in the same group are involved in ribosome biogenesis and eukaryotic translation. The group treated with 5-FU shares similar clusters of upregulated proteins with AG.1 treated group, namely the sub-network of 3 proteins (Apoa1, Apoa2, Dnajc3), while the sub-network of ribosomal constituent proteins is upregulated. The downregulated proteins in this group such as Casp3 and Tlr4 have important roles in apoptosis and immunity, while others, such as S100A9 and Akt1 have roles that are closely entwined with the aforementioned proteins, but also have various other substrates and are important for cell transformation and the progression of colorectal cancer (Dihlmann et al., 2005; Duan et al., 2013). Upregulated and downregulated proteins of the group treated with both AG.1 and 5-FU constitute complex networks of protein-protein interactions with various and multiple functions. Although this group had the largest number of proteins which are differentially regulated, the main biological processes largely overlap with the AG.1 group. Similar to this group, the proteins which are upregulated in this group are involved in lipid metabolism, unfolded protein response and citric acid cycle, while the downregulated proteins are involved in ribosome biogenesis, eukaryotic translation and mRNA processing (splicing).

In order to gain more insight into the biological significance of differentially regulated proteins, REACTOME pathway analysis was performed separately for upregulated and downregulated proteins in each group, according to previously established criteria (**Supplementary Tables 1****–****6** and **Supplementary files**).

### Western Blot

**Figure 5:**
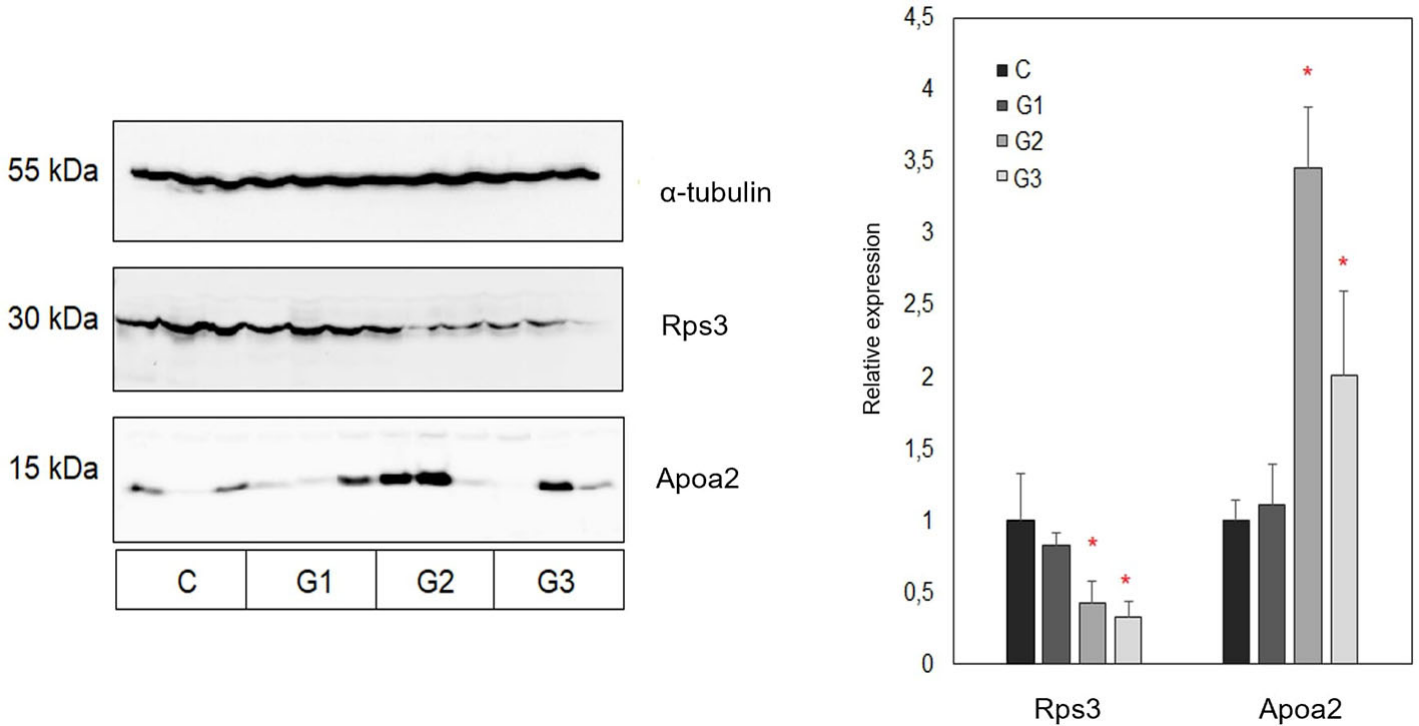
Representative Western blot and summary representation of relative expression of Rps3 and Apoa2 in tumor tissues obtained from control and three treated groups of animals (each group included tumors obtained from three different animals). Results are presented as relative expression values ± SEM between chemiluminescent signals obtained in three replicate experiments. Statistically significant changes (ANOVA, *p* < 0.05) are marked with an asterisk. C, control group of animals; G1, AG.1; G2, AG.1 and 5-FU; G3, 5-FU.

As mass spectrometry results accompanied by the bioinformatics enrichment of identified proteins in analysed tumor tissues revealed several deregulated molecular processes associated with disease progression (including lipid metabolism and the process of translation), in this study the relative expression levels of apolipoprotein A2 (Apoa2) and 40S ribosomal protein S3 (Rps3) were additionally validated by Western blot. Apoa2 is one of the most common high- density lipoproteins mainly associated with the regulation of lipid metabolism (Blanco-Vaca et al., 2001) while Rps3 is a component of the 40S small ribosomal subunit which has an important role in the process of translation (Graifer et al., 2014). In accordance with the results of mass spectrometric profiling of tumor tissue proteomes obtained from the control and treated groups of animals, the results of Western blot analysis have also revealed decreased levels of Rps3 in all treated groups in comparison with the control group (**Figure 5**). Statistical significance was observed for the groups treated with a combination of AG.1 and 5-FU, and 5-FU alone. Furthermore, these observations may indicate decreased protein translation in tumor tissues obtained from treated groups of animals, which is also in line with the results of bioinformatics profiling. In contrast, the Western blot analysis of Apoa2 relative expression has revealed an increase in its relative abundance in tumor tissues obtained from all treated groups of animals when compared to control group. In addition, the most prominent differences were also observed in combined treatment and 5-fluorouracil treated groups in comparison with control group of animals. These observations are also in accordance with the results of proteomic profiling accompanied by the bioinformatics enrichment of identified proteins, which may be indicative for the deregulation of biological processes associated with metabolism of lipids in tumor tissues obtained from treated groups of animals.

## Discussion

Translation, a process of protein synthesis on the basis of a mRNA template, has only recently gained more attention as a fundamental process which is ubiquitously dysregulated during neoplastic transformation and progression. While mostly signaling pathways are in focus for targeted cancer therapies, signal transduction converges on the regulation of the translation machinery, which represents one of the most downstream hubs of signaling cascades (Parsyan, 2014). In eukaryotes especially, this complex and highly choreographed process is performed by a large number of translation factors and involves a host of ribosomal proteins, while its regulation is based on the proliferative, nutritional and other cell states. Of the four phases of protein synthesis (initiation, elongation, termination and ribosome recycling), initiation requires the greatest number of protein factors and is therefore the most common rate-limiting step in this process (Sonenberg and Hinnebusch, 2009). Also, the canonical oncogenic pathways converge on cap-dependent initiation machinery. Various tyrosine kinases and oncoproteins such as MYC and RAS activate eIF4F during malignant proliferation, and oncogenic mutations upregulate various transcription factors thereby promoting further cancer progression. It has been demonstrated that sustained activation of eIF4F in tumors leads to a selective increase in the recruitment of ribosomes to mRNA which encodes growth factors and cytokines (EGF, FGF-2, IGF-2, PDGF, TGF, VEGF, IL-15, Wnt/β-catenin) and various components of receptor signaling cascades such as protein kinases (MOS, PIM1), transcription factors (FOS, MYC), inhibitors of apoptosis (survivin, BCL-2 and BCL-XL), promoters of cell cycle transit (RAS, CDK4 and cyclin D1) as well as enzymes of polyamine synthesis (ornithine decarboxylase and ornithine aminotransferase) (Pond et al., 2010; She et al., 2010; Bitterman and Polunovsky, 2012). Although level of translation is globally decreased in hypoxic tumor tissue, the mRNA of various factors that respond to hypoxia such as HIF-1α, CREB, NF-κB, cyclin-D1, c-MYC, VEGF, and others are preferentially translated in cancer cells, thereby promoting prosurvival, proproliferative and proangiogenic phenotype (Koritzinsky et al., 2006). Hypoxia also promotes tumor metastasis by translational regulation of various proteins important in various phases of this process including EMT such as SNAIL1, SNAIL2, TWIST1, TWIST2, TGF-β, Wnt, and Notch (Lo et al., 2007). While it is known that some tumor suppressors, such as p53 and p27kip which regulate cell cycle progression and apoptosis are also translated as a result of oncogenic eIF4F, the net effect of cap-dependent translation is nevertheless an increase in cell proliferation and viability (Larsson et al., 2007).

Global dysregulation of translation, notably its progressive upregulation, is a key driver in colorectal cancer pathogenesis (Provenzani et al., 2006; Peng et al., 2016). The pathways such as MAPK and PI3K/AKT/mTOR include various important regulators of CRC pathogenesis, such as PIK3CA, K-RAS, BRAF, PTEN, RTK, and others (Cancer Genome Atlas, 2012). The apoptosis and protein synthesis in CRC is affected mainly at the level of translation (Provenzani et al., 2006). The most studied translation factors that function as oncogenes during tumor formation, growth, invasion and metastasis in various cancers include eIF4E and its repressors 4E-BPs, as well as eIF2α, eIF3, eIF2α, eIF5A (Parsyan et al., 2012).

Although translation has until recently been studied mainly at the level of eukaryotic initiation factors (eIFs), there are many studies confirming the role of various ribosomal proteins (RPs) in tumor growth and progression. Ribosomal proteins are a functionally constitutive part of ribosome (Ferreira-Cerca et al., 2005), although many also have extraribosomal functions, such as DNA replication, transcription and repair, RNA splicing and modification, cell growth and proliferation, regulation of apoptosis and development, and cellular transformation (Lai and Xu, 2007). Increased expression of various ribosomal proteins (rpS8, rpL12, rpL23a, rpL27, and rpL30) have been associated with tumor growth (Kondoh et al., 2001). Increased expression of ribosomal protein genes including rpS3, rpS6, rpS8, rpS12 and rpL5 has been reported in colorectal cancer (Pogue-Geile et al., 1991). Recent large-scale bioinformatics analyses demonstrated an upregulation of large scale ribosomal gene expression in adenomas, carcinomas as well as metastases in comparison to normal colonic epithelium (Zhang et al., 2019). It has been proposed that inhibition of rRNA synthesis could likely cause acute perturbation of ribosome biogenesis, which would result in accumulation of free RPs and subsequent activation of p53 and induction of apoptotic cell death, so this mechanism is considered an attractive therapeutic target (Xu et al., 2016).

Our data showed that the pathways such as ribosome assembly (GO:0042255), translation (GO:0006412) and ribosome biogenesis (GO:0042254) for the downregulated proteins in the group treated with AG.1, are significantly enriched (**Table S2**, **File S1**). Since protein synthesis is an early event in colon carcinogenesis (Zhang et al., 2019), this could partly account for the significant difference in survival between curative (50% OS) vs. preventive (100% OS) groups treated with AG.1. Further protein-protein interaction analysis in AG.1 down-accumulated group using STRING revealed significant enrichment for mRNA splicing (GO:0008380) and mRNA processing (GO:0006397) which are, along with translation and influenza life cycle (R-HSA-168255) the three most relevant pathways in a cluster for which the steady rise in protein abundance has been confirmed during CRC progression (Peng et al., 2016). Moreover, proteins with confirmed interactions with annexin A7 (Anxa7) which is down-accumulated in this group have been implicated in colorectal carcinogenesis (**Figure S2**). Cell division cycle 5-like (Cdc5l) protein has been shown to promote hTERT expression and colorectal tumor growth (Li et al., 2017), while metastasis biomarker Park7/DJ-1 is known to promote colon cancer by stimulating Wnt-β-catenin signaling (Zhou et al., 2018). Cdc5l has multiple interactions with a cluster of proteins involved in mRNA splicing (**File S2**, **Figure S2**). In our research, we found the correlation coefficient between the last tumor volume measured and overall survival in all the groups to be *r* = -0.66, which indicates a moderate negative correlation between the two parameters. In cancer clinical trials, overall survival is considered a definitive end point (Driscoll and Rixe, 2009). On the other hand, it has been established that the volume of the primary tumor has no prognostic significance in colorectal cancer (Wolmark et al., 1986; Compton et al., 2000). Moreover, a recent analysis of 1,357 pairs of patients showed that the smaller primary tumor size was significantly associated with worse overall survival (Li et al., 2019). The most common reason for poor survival after chemotherapy treatment is impaired immune reactivity of the host and the presence of rapidly proliferating resistant cells.

While the downregulated proteome profile of group treated with both AG.1 and 5-FU revealed some differences, the most significant enrichment has likewise been observed in specific pathways related to protein synthesis (GO:0006412 translation, GO:0042254, ribosome biogenesis) and mRNA processing/splicing (GO:0006396) (**Table S6**, **File S3**). Parallelism with AG.1 down-accumulated group is also evident in the Anxa7 downregulation. By analysis of macrophage capping protein (Capg) protein-protein interactions, we revealed its interaction partners, such as Cdcl5. Besides the potential role that Cdcl5 has in CRC growth, it is a component of the PRP19-CDC5L complex that forms an integral part of the spliceosome and is required for activating pre-mRNA splicing (Chanarat and Sträßer, 2013). By alanine tRNA ligase (Aars) enrichment analysis we found that its binding partners are implicated in tRNA aminoacylation i.e. translation regulation (**Figure S3**, **File S4**). SH3 domain-binding glutamic acid-rich-like protein 3 (Sh3bgrl3) protein is implicated in cell redox homeostasis and is known to be upregulated in some cancers, and potentially involved in cell resistance to TNF-α induced apoptosis (Berleth et al., 1999). Moreover, its interactions with zinc finger protein 276 (Zpf276) which may be involved in transcriptional regulation, and Rho GDP-dissociation inhibitor 2 (Arhgdib), which regulates regulation of actin cytoskeleton, mediated by Rho family members, are relevant. Namely, deregulation of cell motility is one of the hallmark events in cancer cell invasion and metastasis (Hanahan and Weinberg, 2011) (**Figures S4** and **S5**). Cavin-1 (Ptrf) has a crucial role in caveolae formation and function (Hill et al., 2008). It is proven to be a negative prognostic factor in CRC in the clinic (Protein Atlas, 2020b), although there have also been reports linking it to the suppression of colorectal cancer progression (Wang et al., 2017). Its interactors have a role in transcription by RNA polymerase I and rRNA transcription (**Figure S6**, **File S5**). Among the downregulated proteins in this group we also found GTP-binding protein SAR1a which is known to be upregulated in colorectal cancer in comparison to normal tissues (Kwong et al., 2005), and which interacts with proteins which form COPII or coatomer, a type of vesicle coat protein that transports proteins from the rough endoplasmic reticulum to the Golgi apparatus (**Figure S7**, **File S6**). Upregulated Sec23a, which is a part of a coatomer complex, is however associated with more favourable prognosis (Protein Atlas, 2020a). Downregulated ubiquitin-specific protease 14 (Usp14) could be regarded as a biomarker for cancer invasion and metastasis, since it is known to be upregulated in various cancer types, including colorectal (Shinji et al., 2006). One of the possible explanations is its strong interaction with Usp7, which when upregulated prevents MDM2 self-ubiquitination thereby promoting p53/TP53 ubiquitination and proteasomal degradation (Tang et al., 2006). Besides a large cluster of ribosomal proteins, another downregulated cluster in group treated with both AG.1 and 5-FU is that of heterogeneous ribonucleoproteins (hnRNPs) which are usually upregulated in colorectal cancer, and their primary function, mRNA processing, is a common feature of colorectal cancer progression (**Figure 4F**) (Carpenter et al., 2006; Peng et al., 2016). Peroxiredoxins-2 and -6 are both thiol-specific peroxidases that catalyze the reduction of hydrogen peroxide and organic hydroperoxides to water and alcohols, so their downregulation could contribute to the elevated levels of these ROS which could abrogate specific induction of selective growth of tumor cells by protecting them against oxidative stress, as has been observed in some cancers (Stresing et al., 2013). Both of these enyzmes are typically upregulated in CRC (Feng et al., 2014). HMGB1, which is the most important member of the high mobility group box protein family, is a nuclear protein with different functions in the cell, since it has a role in cancer progression, angiogenesis, invasion, and metastasis development. It has been proposed as an important prognostic factor in CRC, since it correlates with various clinical parameters (Süren et al., 2014). Serpina1 is the upregulated protein in this group which has a positive prognostic significance in colorectal cancer, and this could result, at least partly, from its function of PTEN stabilization, and/or negative regulation of PI3K/AKT network (Protein Atlas, 2020c).

Besides its known role as an antimetabolite class drug with primary effects on replication and DNA integrity, 5-FU has been proven to influence the proteome of colorectal cancer cells, causing post-translational reduction in abundance of many ribosomal proteins and a downregulation of the translational capacity of the cells (Marin-Vicente et al., 2013). Our results show an enriched translation pathway (GO:0006412) for the upregulated proteins of the group treated with 5-fluorouracil, which seemingly contradicts these findings (**Figure 4C**, **Table S3**). However, this could stem from the fact that the 5-FU has rapid effect on the translation machinery, so this upregulation could point to activation of subsequent extraribosomal functions, such as DNA-damage response (Yang et al., 2018). Furthermore, it was shown previously that 5-FU treatment increased the fraction of ribosome-free L5, L11, and L23 ribosomal proteins and their interaction with MDM2, leading to p53 activation and G1/S arrest (Sun et al., 2007). In the clinic however, various upregulated ribosomal proteins (rpS and rpLs) were overrepresented in non-responders (NR) in comparison to total responders (TR) to 5-fluorouracil treatment, along with other proteins such as DPYD (dihydropyrimidine dehydrogenase) and TYMP (thymidine phosphorylase), which have a role in hepatic metabolism and the transformation of 5-FU into FdUMP, respectively. It was concluded that a higher representation of ribosomal proteins, as well as certain mitochondrial proteins in NR patients’ tumor proteome may be responsible for a lower response to 5-FU treatment, and a potential new mechanism of resistance to 5-FU (Chauvin et al., 2018). Prothymosin-α, which is upregulated in the 5-FU group, has been linked to poor prognosis in colorectal cancer patients, and its interactors are primarily involved in transcription regulation *via* chromatin remodeling (**Figure S8**, **File S7**) (Zhang et al., 2014). It has been established that caveolins are involved in both tumor suppression and oncogenesis, depending on tumor type and progress stage. High expression of caveolins and cavins leads to inhibition of cancer-related pathways such as growth factor signaling pathways (Gupta et al., 2014). Cavin-2 or caveolae-associated protein (Sdpr), which we found to be upregulated in both AG.1 and 5-FU treated groups, interacts with Cavin-1 (Ptrf) and both have a role in suppressing the progression and metastasis of colorectal cancers. Ptrf downregulation correlates to the more advanced stage of the disease in the clinic (Wang et al., 2017). Cyclin-dependent kinase 11B (Cdk11b) has been found upregulated in all three treated groups. It has been observed that Cdk11 is ubiquitously expressed in human cancer cell lines, and it has been found to be a positive modulator of Wnt/β-catenin pathway in colon cancer (Zhou et al., 2016). The elevated level of importin-7 is in accordance with the upregulated ribosomal proteins in this group, since it has been found that it participates in the activation of nuclear import of ribosomal proteins, i.e., in ribosome biogenesis. Importin-7 is elevated in cancer, and it is believed that when ribosome biogenesis is disrupted, unassembled ribosomal proteins are released from nucleolar ribosome assembly “factories” and are then free to bind Mdm2 and activate p53 (Golomb et al., 2012). Metallothionein-1 is up-accumulated in 5-FU treated group. Its downregulation in colorectal cancer has been linked with lower survival in CRC, so its positive prognostic significance could be linked to the previously observed effect of its elevated levels and induced differentiation of colorectal cancer cells (Arriaga et al., 2017). Proteins that were downregulated in this group include S100A9, which is often co-expressed with S100A8, and both are calcium binding proteins, which exert their effects through MAPK and NF-κB pathways activation. Their overexpression has been associated with carcinogenesis of various tumors, including CRC (Stulík et al., 1999). Pathways that were enriched for the downregulated proteins are mostly involved in positive regulation of apoptotic process (GO:0043065) and regulation of TLR by endogenous ligand (R-HSA-5686938) i.e. immune system process (GO:0002376), which indicates an immunosuppressive effect of 5-FU in the advanced cancer stages (**Table S4**, **File S8**). Immunosuppressive effect of 5-fluorouracil is the result of severe bone marrow suppression and is one of the most common toxicities observed with its prolonged use, especially with bolus schedules (Macdonald, 1999).

Unfolded protein response (UPR) is a conserved mechanism of cellular stress response related to the endoplasmic reticulum stress, and has a complex role in cancer, since each of the canonical functions of UPR can serve as a mechanism that can limit or facilitate tumorigenesis. It has been shown that acute UPR signaling inhibits translation, induces chaperone expression, and activates proteolysis (ER-associated degradation system by which misfolded proteins are removed from ER), while chronic UPR signaling can lead to apoptosis (Clarke, 2019). In case of colorectal cancer, it has been reported recently that the activation of the unfolded protein response causes differentiation of colon cancer stem cells, which enhances their responses to chemotherapy *in vitro* and *in vivo* (Wielenga et al., 2015). Another group showed that activation of either of the three canonical UPR pathways, PERK, ATF6, or XBP1 results in reduced cellular proliferation and reduced expression of markers of intestinal epithelial stemness, whereas IRE1-XBP1 and ATF6 activation also reduced global protein synthesis (Spaan et al., 2019). Moreover, by analyzing the dynamics of differentially regulated proteins during colorectal cancer carcinogenesis, Peng et al. showed that the unfolded protein response was mostly downregulated during CRC progression, which is in accordance with this research. Our results indicate that the unfolded protein stress response is elevated in all of the treatment groups (**Tables S1**, **S3**, **S5**), indicated by the DnaJ homolog subfamily C member 3 (Dnajc3) and its interactors up-accumulation. It has been proven that Dnajc3 upregulation is triggered by the IRE1 arm of the UPR response, and the heat shock protein 90 (HSP90) is necessary for IRE1α and PERK stability (Marcu et al., 2002; Pearse and Hebert, 2006) (**Figure S9**).

The pathway that was enriched in all upregulated treatment groups in comparison to control was regulation of lipid metabolism by peroxisome proliferator-activated receptor alpha (PPAR-α) (**Tables S1**, **S3**, and **S5**). PPAR-α is a nuclear receptor that regulates systemic lipid homeostasis, cell proliferation, differentiation, energy metabolism, oxidative stress, inflammation, circadian rhythms, immune response and cell differentiation. PPAR-α agonists such as fenofibrate are used to treat hyperlipidemia and are also known to have anticancer effects (Morinishi et al., 2019). It has been demonstrated previously that 5-fluorouracil also exerts its anticancer effects by lipid metabolism-related factors such as increase in PPAR-γ expression (Yu et al., 2005). Human colorectal tumors have lower levels of PPARA mRNA and protein than nontumor tissues and loss of PPAR-α promotes colon carcinogenesis by increasing DNMT1 methyltransferase mediated methylation of p21 and PRMT6 methyltransferase mediated methylation of p27 (Luo et al., 2019). Activation of PPAR-α promotes uptake, utilization, and catabolism of fatty acids by upregulation of genes involved in fatty acid transport, fatty acid binding and activation, and peroxisomal and mitochondrial fatty acid β-oxidation (Kersten, 2014). In general, fatty acid oxidation has been proven to be downregulated in multiple tumors, and its activation is associated with lowered cancer cell proliferation and improved outcomes in some cancers (Aiderus et al., 2018).

One of the key pathways that were enriched in all three upregulated groups in comparison to control is the tricarboxylic acid (TCA) cycle, which is a central route for oxidative phosphorylation in cells (**Tables S1**, **S3**, and **S5**). It is well known that while glucose provides the main source of pyruvate entering the TCA in normal cells, cancer cells often shunt glucose away from the TCA cycle for catabolism through anaerobic glycolysis. These cells are then more dependent on glutamine and fatty acids to replenish TCA cycle intermediates (Eagle, 1955). Fatty acid β-oxidation produces acetyl-CoA which enters the TCA cycle to generate NADH and FADH2 for the electron transport chain which ultimately serves to synthesis of approximately six times more ATP than oxidation of carbohydrates (Koundouros and Poulogiannis, 2020). This metabolic reprogramming known as the Warburg effect, which serves to support cancer cell proliferation, growth and dissemination has been recognized as a pivotal hallmark of cancer (Hanahan and Weinberg, 2011). Semenza reported that HIF activation, which is characteristic for the tumor hypoxic microenvironment, orchestrates a metabolic program that promotes the catabolism of glucose through aerobic glycolysis and thus shifts glucose away from the TCA cycle (Semenza, 2012). One of the roles of wild-type TP53 in metabolism is lowering the rates of glycolysis and promoting oxidative phosphorylation (Anderson et al., 2018). Further indication of TCA activation is provided by the upregulation of fumarate hydratase (Fh) in all treatment groups, as well as the electron transfer flavoprotein (Etf) cluster in the group treated with both AG.1 and 5-FU, which are involved in oxidative phosphorylation (**Figure 4E**). Fh is an enzyme necessary for fumarate to malate conversion in the TCA cycle, and its deficiency or loss is associated with increased incidence of various cancers, including CRC, due to fumarate accumulation (Hu et al., 2013). Fh deficient cells undergo metabolic rewiring since combined disruption of the TCA cycle and the inhibition of succinate dehydrogenase (Complex II of the respiratory chain) by fumarate significantly reduces mitochondrial respiration (Schmidt et al., 2019). This is paralleled by an increase in their glycolytic rates and by shunting glucose into lactate production and other glycolytic branches, such as pentose phosphate pathway (PPP) instead of oxidizing it in the mitochondria. Moreover, it has been reported that Fh deficient cancer cells increase their fatty acid synthesis by diminishing phosphorylation of acetyl CoA carboxylase, a rate-limiting step in this process (Anderson et al., 2018). Besides increased lipid biosynthesis, Fh loss increases protein synthesis through mTOR upregulation as well as DNA damage response and repair. Increased fumarate and decreased iron levels in Fh-deficient cancer cells inactivate prolyl hydroxylases, leading to stabilization of hypoxia-inducible factor (HIF)-1α and increased expression of genes such as VEGF and glucose transporter 1 (GLUT1) to provide fuel needed for rapid growth demands. Further oncogenic processes which are upregulated by Fh loss include EMT and epigenetic reprogramming, which includes DNA and histone hypermethylation, which confirm fumarate hydratase to be a crucial tumor suppressor (Schmidt et al., 2019). The role of TCA cycle in CRC progression has been established, since it is known that the abundance of proteins associated with this process decreases progressively through adenoma-carcinoma *in situ*-invasive colorectal carcinoma sequence (Peng et al., 2016).

In summary, our study has revealed for the first time the anticancer effects of medicinal mushroom complex extract mixture by using high-throughput TMT quantitative proteomics of tumor tissues. Recent large-scale proteomic research has revealed differentially expressed proteins during multistage carcinogenesis from normal colon, adenoma, carcinoma *in situ* to invasive carcinoma human tissues (Peng et al., 2016; Zhang et al., 2019). This has enabled the temporal observation of large-scale processes important in CRC progression, as well as a detailed insight into function of many biomarkers of prognostic significance in the clinic.

This study has shown that AG.1 alone, which is a complex extract mixture of six well characterized and safe medicinal mushroom species, as well as in combination with known antimetabolite class drug 5-FU, evoke changes contrary to those found in colorectal carcinogenesis, which result in significantly improved survival. These antitumor effects are associated with the shift in energy production pathways, pointing to the increased lipid metabolism and tricarboxylic acid cycle (TCA) as well to the upregulated unfolded protein response (UPR). Importantly, by performing bioinformatic analysis we found that the host of DEPs which are down-accumulated are involved in ribosomal biogenesis, translation, influenza life cycle, and mRNA processing/splicing which points to an additional mechanism of antitumor action of the studied preparation with or without 5-fluorouracil, since these processes are significantly upregulated during CRC progression. Differentially regulated proteins involved in the processes of lipid metabolism and translation, Apoa2 and Rps3 respectively, have been further validated by Western blot analysis. Because the genes which code for these processes are evolutionary conserved, these insights warrant further translational research, in light of the need to develop new scientifically verified biotherapies of cancer.

## Data Availability Statement

The datasets presented in this study can be found in online repositories. The names of the repository/repositories and accession number(s) can be found below: https://www.ebi.ac.uk/pride/archive/, PXD018827.

## Ethics Statement

The animal study was reviewed and approved by the University’s of Zagreb, Department of Biology Ethics Committee (approval code: 251-58-10617-16-14).

## Author Contributions

Conceptualization: SK and NO. Methodology: SK, NO, BJ, and IJ. Formal analysis: AG, BJ, and MK. Investigation: BJ, AH, MK, and PG. Writing—original draft preparation: BJ, MK, AH, and AG. Writing—review and editing: BJ, SK, NO, IJ, AH, AG, and MK. Visualization, BJ and MK. Supervision, SK and NO. Funding acquisition, SK and IJ. All authors contributed to the article and approved the submitted version.

## Funding

The study was funded by the University of Rijeka research grant uniri-biomed-18-133 given to SKP and co-funded (reagents supply) by Dr Myko San Co., Zagreb, Croatia.

## Conflict of Interest

BJ and IJ are employees of Dr Myko San Co. and have not been involved in the conceptualization. BJ performed investigation, analysis and interpretation of data, and was involved in manuscript writing, as part of his PhD. IJ has been involved in manuscript review and editing.

The remaining authors declare that the research was conducted in the absence of any commercial or financial relationships that could be construed as a potential conflict of interest.
