# Supplementary material for: Treatment With Medicinal Mushroom Extract Mixture Inhibits Translation and Reprograms Metabolism in Advanced Colorectal Cancer Animal Model as Evidenced by Tandem Mass Tags Proteomics Analysis
Source: Front Pharmacol. 2020 Aug 21;11:1202. doi: 10.3389/fphar.2020.01202 (PMC7472604; doi:10.3389/fphar.2020.01202)
Supplement: Supplementary file 9 [file DataSheet_9.docx]

**Supplementary tables**

**Supplementary table 1.** Enriched REACTOME pathways for up-accumulated proteins of the Agarikon.1 treated group

| Pathway identifier | Pathway name | #Entities found | #Entities total | #Interactors found | #Interactors total | Entities ratio | Entities pValue | Entities FDR |
| --- | --- | --- | --- | --- | --- | --- | --- | --- |
| R-HSA-8957275 | Post-translational protein phosphorylation | 6 | 109 | 0 | 0 | 0.007552 | 1.68E-06 | 0.00128 |
| R-HSA-71403 | Citric acid cycle (TCA cycle) | 5 | 50 | 0 | 32 | 0.003464 | 7.03E-06 | 0.002686 |
| R-HSA-8963888 | Chylomicron assembly | 4 | 14 | 1 | 37 | 9.70E-04 | 2.02E-05 | 0.005133 |
| R-HSA-1989781 | PPARA activates gene expression | 9 | 174 | 3 | 342 | 0.012056 | 3.83E-05 | 0.007306 |
| R-HSA-400206 | Regulation of lipid metabolism by PPARalpha | 9 | 176 | 3 | 372 | 0.012194 | 5.62E-05 | 0.008548 |
| R-HSA-381426 | Regulation of Insulin-like Growth Factor (IGF) transport and uptake by Insulin-like Growth Factor Binding Proteins (IGFBPs) | 6 | 127 | 0 | 103 | 0.008799 | 1.01E-04 | 0.012866 |
| R-HSA-71406 | Pyruvate metabolism and Citric Acid (TCA) cycle | 5 | 98 | 0 | 67 | 0.00679 | 1.90E-04 | 0.020658 |
| R-HSA-2187338 | Visual phototransduction | 6 | 169 | 0 | 111 | 0.011709 | 2.68E-04 | 0.025418 |
| R-HSA-8963898 | Plasma lipoprotein assembly | 4 | 30 | 1 | 75 | 0.002079 | 3.61E-04 | 0.026764 |
| R-HSA-975634 | Retinoid metabolism and transport | 4 | 79 | 0 | 33 | 0.005474 | 4.32E-04 | 0.026764 |
| R-HSA-381183 | ATF6 (ATF6-alpha) activates chaperone genes | 4 | 15 | 3 | 99 | 0.001039 | 4.63E-04 | 0.026764 |
| R-HSA-8963901 | Chylomicron remodeling | 3 | 17 | 0 | 30 | 0.001178 | 4.81E-04 | 0.026764 |
| R-HSA-381033 | ATF6 (ATF6-alpha) activates chaperones | 4 | 17 | 3 | 99 | 0.001178 | 4.96E-04 | 0.026764 |
| R-HSA-6806667 | Metabolism of fat-soluble vitamins | 4 | 94 | 0 | 112 | 0.006513 | 0.00421 | 0.210499 |
| R-HSA-381119 | Unfolded Protein Response (UPR) | 8 | 155 | 4 | 697 | 0.010739 | 0.005648 | 0.265478 |
| R-HSA-1428517 | The citric acid (TCA) cycle and respiratory electron transport | 5 | 229 | 1 | 209 | 0.015866 | 0.008946 | 0.380712 |
| R-HSA-8963899 | Plasma lipoprotein remodeling | 3 | 54 | 1 | 81 | 0.003741 | 0.009137 | 0.380712 |
| R-HSA-445355 | Smooth Muscle Contraction | 3 | 55 | 0 | 80 | 0.003811 | 0.009518 | 0.380712 |
| R-HSA-3000480 | Scavenging by Class A Receptors | 2 | 48 | 0 | 0 | 0.003326 | 0.010888 | 0.399402 |
| R-HSA-174824 | Plasma lipoprotein assembly, remodeling, and clearance | 4 | 98 | 2 | 179 | 0.00679 | 0.011094 | 0.399402 |
| R-HSA-1369062 | ABC transporters in lipid homeostasis | 2 | 29 | 0 | 24 | 0.002009 | 0.011765 | 0.399997 |
| R-HSA-381070 | IRE1alpha activates chaperones | 5 | 101 | 1 | 349 | 0.006998 | 0.015106 | 0.464752 |
| R-HSA-381340 | Transcriptional regulation of white adipocyte differentiation | 5 | 108 | 1 | 400 | 0.007483 | 0.020723 | 0.464752 |
| R-HSA-5682113 | Defective ABCA1 causes Tangier disease | 1 | 5 | 0 | 2 | 3.46E-04 | 0.022546 | 0.464752 |
| R-HSA-6796648 | TP53 Regulates Transcription of DNA Repair Genes | 3 | 89 | 3 | 115 | 0.006166 | 0.026377 | 0.464752 |
| R-HSA-2453902 | The canonical retinoid cycle in rods (twilight vision) | 2 | 57 | 0 | 28 | 0.003949 | 0.030944 | 0.464752 |
| R-HSA-425381 | Bicarbonate transporters | 1 | 14 | 0 | 1 | 9.70E-04 | 0.0477 | 0.464752 |
| R-HSA-2514853 | Condensation of Prometaphase Chromosomes | 1 | 15 | 0 | 0 | 0.001039 | 0.0477 | 0.464752 |
| R-HSA-69091 | Polymerase switching | 1 | 16 | 0 | 0 | 0.001109 | 0.050799 | 0.464752 |

**Supplementary table 2.** Enriched REACTOME pathways for down-accumulated proteins of the Agarikon.1 treated group

| Pathway identifier | Pathway name | #Entities found | #Entities total | #Interactors found | #Interactors total | Entities ratio | Entities pValue | Entities FDR |
| --- | --- | --- | --- | --- | --- | --- | --- | --- |
| R-HSA-72689 | Formation of a pool of free 40S subunits | 8 | 106 | 0 | 6 | 0.007344 | 4.56E-13 | 7.18E-11 |
| R-HSA-72764 | Eukaryotic Translation Termination | 8 | 106 | 0 | 6 | 0.007344 | 4.90E-13 | 7.18E-11 |
| R-HSA-2408557 | Selenocysteine synthesis | 8 | 113 | 0 | 2 | 0.007829 | 6.48E-13 | 7.18E-11 |
| R-HSA-1799339 | SRP-dependent cotranslational protein targeting to membrane | 8 | 119 | 0 | 0 | 0.008245 | 8.50E-13 | 7.18E-11 |
| R-HSA-975956 | Nonsense Mediated Decay (NMD) independent of the Exon Junction Complex (EJC) | 8 | 101 | 0 | 23 | 0.006998 | 9.70E-13 | 7.18E-11 |
| R-HSA-156827 | L13a-mediated translational silencing of Ceruloplasmin expression | 8 | 120 | 0 | 2 | 0.008314 | 1.03E-12 | 7.18E-11 |
| R-HSA-156902 | Peptide chain elongation | 8 | 97 | 0 | 28 | 0.006721 | 1.18E-12 | 7.18E-11 |
| R-HSA-72702 | Ribosomal scanning and start codon recognition | 7 | 64 | 0 | 8 | 0.004434 | 1.42E-12 | 7.53E-11 |
| R-HSA-192823 | Viral mRNA Translation | 8 | 114 | 0 | 22 | 0.007899 | 2.31E-12 | 1.08E-10 |
| R-HSA-72695 | Formation of the ternary complex, and subsequently, the 43S complex | 7 | 54 | 0 | 24 | 0.003741 | 3.69E-12 | 1.59E-10 |
| R-HSA-72706 | GTP hydrolysis and joining of the 60S ribosomal subunit | 8 | 120 | 0 | 47 | 0.008314 | 7.60E-12 | 2.96E-10 |
| R-HSA-72649 | Translation initiation complex formation | 7 | 62 | 0 | 32 | 0.004296 | 9.95E-12 | 3.48E-10 |
| R-HSA-156842 | Eukaryotic Translation Elongation | 8 | 102 | 0 | 67 | 0.007067 | 1.07E-11 | 3.54E-10 |
| R-HSA-927802 | Nonsense-Mediated Decay (NMD) | 8 | 124 | 0 | 77 | 0.008591 | 2.89E-11 | 8.09E-10 |
| R-HSA-975957 | Nonsense Mediated Decay (NMD) enhanced by the Exon Junction Complex (EJC) | 8 | 124 | 0 | 77 | 0.008591 | 2.89E-11 | 8.09E-10 |
| R-HSA-2408522 | Selenoamino acid metabolism | 8 | 181 | 0 | 15 | 0.012541 | 4.36E-11 | 1.13E-09 |
| R-HSA-72737 | Cap-dependent Translation Initiation | 8 | 130 | 0 | 106 | 0.009007 | 1.16E-10 | 2.76E-09 |
| R-HSA-72613 | Eukaryotic Translation Initiation | 8 | 130 | 0 | 107 | 0.009007 | 1.20E-10 | 2.76E-09 |
| R-HSA-72662 | Activation of the mRNA upon binding of the cap-binding complex and eIFs, and subsequent binding to 43S | 7 | 66 | 0 | 76 | 0.004573 | 1.41E-10 | 3.10E-09 |
| R-HSA-168273 | Influenza Viral RNA Transcription and Replication | 8 | 175 | 0 | 88 | 0.012125 | 3.23E-10 | 6.79E-09 |
| R-HSA-9010553 | Regulation of expression of SLITs and ROBOs | 8 | 183 | 0 | 84 | 0.012679 | 4.00E-10 | 8.00E-09 |
| R-HSA-9633012 | Response of EIF2AK4 (GCN2) to amino acid deficiency | 8 | 115 | 0 | 165 | 0.007968 | 6.20E-10 | 1.18E-08 |
| R-HSA-6791226 | Major pathway of rRNA processing in the nucleolus and cytosol | 9 | 189 | 2 | 377 | 0.013095 | 4.68E-09 | 8.43E-08 |
| R-HSA-8868773 | rRNA processing in the nucleus and cytosol | 9 | 207 | 2 | 382 | 0.014342 | 6.65E-09 | 1.13E-07 |
| R-HSA-168255 | Influenza Life Cycle | 8 | 188 | 0 | 224 | 0.013026 | 9.93E-09 | 1.69E-07 |
| R-HSA-72312 | rRNA processing | 9 | 245 | 2 | 425 | 0.016975 | 2.13E-08 | 3.40E-07 |
| R-HSA-168254 | Influenza Infection | 8 | 199 | 1 | 264 | 0.013788 | 2.29E-08 | 3.44E-07 |
| R-HSA-376176 | Signaling by ROBO receptors | 8 | 235 | 0 | 326 | 0.016282 | 1.14E-07 | 1.71E-06 |
| R-HSA-72766 | Translation | 8 | 339 | 0 | 345 | 0.023488 | 5.33E-07 | 7.46E-06 |
| R-HSA-71291 | Metabolism of amino acids and derivatives | 8 | 660 | 0 | 273 | 0.045729 | 5.98E-06 | 8.37E-05 |
| R-HSA-2262752 | Cellular responses to stress | 9 | 690 | 0 | 1072 | 0.047807 | 5.88E-05 | 7.65E-04 |
| R-HSA-8953897 | Cellular responses to external stimuli | 9 | 708 | 0 | 1090 | 0.049054 | 6.90E-05 | 8.97E-04 |
| R-HSA-8953854 | Metabolism of RNA | 10 | 782 | 3 | 1643 | 0.054181 | 8.57E-05 | 0.001114 |
| R-HSA-422475 | Axon guidance | 8 | 584 | 1 | 1478 | 0.040463 | 0.001142 | 0.013702 |
| R-HSA-5663205 | Infectious disease | 8 | 801 | 2 | 1881 | 0.055498 | 0.006193 | 0.074312 |
| R-HSA-169131 | Inhibition of PKR | 0 | 2 | 1 | 24 | 1.39E-04 | 0.028765 | 0.248142 |
| R-HSA-450321 | JNK (c-Jun kinases) phosphorylation and activation mediated by activated human TAK1 | 1 | 26 | 0 | 0 | 0.001801 | 0.029899 | 0.248142 |
| R-HSA-418359 | Reduction of cytosolic Ca++ levels | 1 | 15 | 1 | 13 | 0.001039 | 0.031031 | 0.248142 |
| R-HSA-425561 | Sodium/Calcium exchangers | 1 | 15 | 1 | 14 | 0.001039 | 0.032163 | 0.248142 |
| R-HSA-5603027 | IKBKG deficiency causes anhidrotic ectodermal dysplasia with immunodeficiency (EDA-ID) (via TLR) | 0 | 3 | 1 | 37 | 2.08E-04 | 0.042288 | 0.248142 |
| R-HSA-1266738 | Developmental Biology | 8 | 1207 | 2 | 2723 | 0.083628 | 0.046459 | 0.248142 |
| R-HSA-5626978 | TNFR1-mediated ceramide production | 0 | 6 | 1 | 36 | 4.16E-04 | 0.04787 | 0.248142 |
| R-HSA-5684264 | MAP3K8 (TPL2)-dependent MAPK1/3 activation | 0 | 18 | 1 | 34 | 0.001247 | 0.050094 | 0.248142 |
| R-HSA-209560 | NF-kB is activated and signals survival | 0 | 15 | 1 | 37 | 0.001039 | 0.05342 | 0.248142 |

**Supplementary table 3.** Enriched REACTOME pathways for up-accumulated proteins of the 5-fluorouracil treated group

| Pathway identifier | Pathway name | #Entities found | #Entities total | #Interactors found | #Interactors total | Entities ratio | Entities pValue | Entities FDR |
| --- | --- | --- | --- | --- | --- | --- | --- | --- |
| R-HSA-192823 | Viral mRNA Translation | 7 | 114 | 0 | 22 | 0.007899 | 3.03E-06 | 0.001385 |
| R-HSA-1989781 | PPARA activates gene expression | 12 | 174 | 2 | 342 | 0.012056 | 3.15E-06 | 0.001385 |
| R-HSA-400206 | Regulation of lipid metabolism by PPARalpha | 12 | 176 | 2 | 372 | 0.012194 | 5.24E-06 | 0.001534 |
| R-HSA-72706 | GTP hydrolysis and joining of the 60S ribosomal subunit | 7 | 120 | 0 | 47 | 0.008314 | 8.06E-06 | 0.001558 |
| R-HSA-8957275 | Post-translational protein phosphorylation | 6 | 109 | 0 | 0 | 0.007552 | 1.14E-05 | 0.001558 |
| R-HSA-72689 | Formation of a pool of free 40S subunits | 6 | 106 | 0 | 6 | 0.007344 | 1.20E-05 | 0.001558 |
| R-HSA-72764 | Eukaryotic Translation Termination | 6 | 106 | 0 | 6 | 0.007344 | 1.26E-05 | 0.001558 |
| R-HSA-2408557 | Selenocysteine synthesis | 6 | 113 | 0 | 2 | 0.007829 | 1.54E-05 | 0.001558 |
| R-HSA-1799339 | SRP-dependent cotranslational protein targeting to membrane | 6 | 119 | 0 | 0 | 0.008245 | 1.86E-05 | 0.001558 |
| R-HSA-975956 | Nonsense Mediated Decay (NMD) independent of the Exon Junction Complex (EJC) | 6 | 101 | 0 | 23 | 0.006998 | 2.04E-05 | 0.001558 |
| R-HSA-168273 | Influenza Viral RNA Transcription and Replication | 8 | 175 | 1 | 88 | 0.012125 | 2.11E-05 | 0.001558 |
| R-HSA-156827 | L13a-mediated translational silencing of Ceruloplasmin expression | 6 | 120 | 0 | 2 | 0.008314 | 2.13E-05 | 0.001558 |
| R-HSA-156902 | Peptide chain elongation | 6 | 97 | 0 | 28 | 0.006721 | 2.34E-05 | 0.001565 |
| R-HSA-72737 | Cap-dependent Translation Initiation | 7 | 130 | 0 | 106 | 0.009007 | 7.18E-05 | 0.003921 |
| R-HSA-8963888 | Chylomicron assembly | 4 | 14 | 1 | 37 | 9.70E-04 | 7.33E-05 | 0.003921 |
| R-HSA-72613 | Eukaryotic Translation Initiation | 7 | 130 | 0 | 107 | 0.009007 | 7.39E-05 | 0.003921 |
| R-HSA-168255 | Influenza Life Cycle | 9 | 188 | 2 | 224 | 0.013026 | 7.69E-05 | 0.003921 |
| R-HSA-156842 | Eukaryotic Translation Elongation | 6 | 102 | 0 | 67 | 0.007067 | 1.08E-04 | 0.005195 |
| R-HSA-156588 | Glucuronidation | 4 | 55 | 0 | 2 | 0.003811 | 1.41E-04 | 0.006505 |
| R-HSA-168254 | Influenza Infection | 9 | 199 | 3 | 264 | 0.013788 | 1.73E-04 | 0.007433 |
| R-HSA-927802 | Nonsense-Mediated Decay (NMD) | 6 | 124 | 0 | 77 | 0.008591 | 2.13E-04 | 0.008298 |
| R-HSA-975957 | Nonsense Mediated Decay (NMD) enhanced by the Exon Junction Complex (EJC) | 6 | 124 | 0 | 77 | 0.008591 | 2.13E-04 | 0.008298 |
| R-HSA-2408522 | Selenoamino acid metabolism | 6 | 181 | 0 | 15 | 0.012541 | 2.81E-04 | 0.01068 |
| R-HSA-381426 | Regulation of Insulin-like Growth Factor (IGF) transport and uptake by Insulin-like Growth Factor Binding Proteins (IGFBPs) | 6 | 127 | 0 | 103 | 0.008799 | 6.07E-04 | 0.021842 |
| R-HSA-9010553 | Regulation of expression of SLITs and ROBOs | 6 | 183 | 0 | 84 | 0.012679 | 0.001217 | 0.039958 |
| R-HSA-8963898 | Plasma lipoprotein assembly | 4 | 30 | 1 | 75 | 0.002079 | 0.001243 | 0.039958 |
| R-HSA-8963901 | Chylomicron remodeling | 3 | 17 | 0 | 30 | 0.001178 | 0.001249 | 0.039958 |
| R-HSA-3371568 | Attenuation phase | 3 | 47 | 0 | 1 | 0.003256 | 0.00141 | 0.042286 |
| R-HSA-5660526 | Response to metal ions | 3 | 21 | 0 | 27 | 0.001455 | 0.00141 | 0.042286 |
| R-HSA-381183 | ATF6 (ATF6-alpha) activates chaperone genes | 4 | 15 | 3 | 99 | 0.001039 | 0.001582 | 0.045208 |
| R-HSA-9633012 | Response of EIF2AK4 (GCN2) to amino acid deficiency | 6 | 115 | 1 | 165 | 0.007968 | 0.001615 | 0.045208 |
| R-HSA-381033 | ATF6 (ATF6-alpha) activates chaperones | 4 | 17 | 3 | 99 | 0.001178 | 0.00169 | 0.045626 |
| R-HSA-3371571 | HSF1-dependent transactivation | 3 | 59 | 0 | 1 | 0.004088 | 0.002648 | 0.068841 |
| R-HSA-8941856 | RUNX3 regulates NOTCH signaling | 2 | 16 | 1 | 11 | 0.001109 | 0.005872 | 0.146793 |
| R-HSA-381340 | Transcriptional regulation of white adipocyte differentiation | 7 | 108 | 4 | 400 | 0.007483 | 0.006433 | 0.160832 |
| R-HSA-2187338 | Visual phototransduction | 5 | 169 | 0 | 111 | 0.011709 | 0.00804 | 0.192958 |
| R-HSA-8953897 | Cellular responses to external stimuli | 15 | 708 | 6 | 1090 | 0.049054 | 0.008495 | 0.195379 |
| R-HSA-975634 | Retinoid metabolism and transport | 3 | 79 | 0 | 33 | 0.005474 | 0.012902 | 0.283837 |
| R-HSA-5661231 | Metallothioneins bind metals | 2 | 16 | 0 | 24 | 0.001109 | 0.014388 | 0.302149 |
| R-HSA-210744 | Regulation of gene expression in late stage (branching morphogenesis) pancreatic bud precursor cells | 2 | 20 | 0 | 22 | 0.001386 | 0.015771 | 0.331187 |
| R-HSA-3000480 | Scavenging by Class A Receptors | 2 | 48 | 0 | 0 | 0.003326 | 0.020243 | 0.404856 |
| R-HSA-1369062 | ABC transporters in lipid homeostasis | 2 | 29 | 0 | 24 | 0.002009 | 0.021838 | 0.416525 |
| R-HSA-8963899 | Plasma lipoprotein remodeling | 3 | 54 | 1 | 81 | 0.003741 | 0.021922 | 0.416525 |
| R-HSA-72766 | Translation | 7 | 339 | 0 | 345 | 0.023488 | 0.030721 | 0.525 |
| R-HSA-5682113 | Defective ABCA1 causes Tangier disease | 1 | 5 | 0 | 2 | 3.46E-04 | 0.031158 | 0.525 |
| R-HSA-174824 | Plasma lipoprotein assembly, remodeling, and clearance | 4 | 98 | 2 | 179 | 0.00679 | 0.032595 | 0.525 |
| R-HSA-6791226 | Major pathway of rRNA processing in the nucleolus and cytosol | 6 | 189 | 0 | 377 | 0.013095 | 0.033682 | 0.525 |
| R-HSA-2644602 | Signaling by NOTCH1 PEST Domain Mutants in Cancer | 4 | 68 | 2 | 212 | 0.004711 | 0.034148 | 0.525 |
| R-HSA-2894862 | Constitutive Signaling by NOTCH1 HD+PEST Domain Mutants | 4 | 68 | 2 | 212 | 0.004711 | 0.034148 | 0.525 |
| R-HSA-2644606 | Constitutive Signaling by NOTCH1 PEST Domain Mutants | 4 | 68 | 2 | 212 | 0.004711 | 0.034148 | 0.525 |
| R-HSA-2894858 | Signaling by NOTCH1 HD+PEST Domain Mutants in Cancer | 4 | 68 | 2 | 212 | 0.004711 | 0.034148 | 0.525 |
| R-HSA-2644603 | Signaling by NOTCH1 in Cancer | 4 | 68 | 2 | 212 | 0.004711 | 0.034148 | 0.525 |
| R-HSA-381119 | Unfolded Protein Response (UPR) | 8 | 155 | 5 | 697 | 0.010739 | 0.035363 | 0.525 |
| R-HSA-9636249 | Inhibition of nitric oxide production | 1 | 5 | 0 | 3 | 3.46E-04 | 0.03553 | 0.525 |
| R-HSA-376176 | Signaling by ROBO receptors | 6 | 235 | 0 | 326 | 0.016282 | 0.036093 | 0.525 |
| R-HSA-3371556 | Cellular response to heat stress | 4 | 135 | 1 | 157 | 0.009354 | 0.038216 | 0.525 |
| R-HSA-72702 | Ribosomal scanning and start codon recognition | 2 | 64 | 0 | 8 | 0.004434 | 0.038344 | 0.525 |
| R-HSA-8868773 | rRNA processing in the nucleus and cytosol | 6 | 207 | 0 | 382 | 0.014342 | 0.039769 | 0.525 |
| R-HSA-5660489 | MTF1 activates gene expression | 1 | 6 | 0 | 3 | 4.16E-04 | 0.039882 | 0.525 |
| R-HSA-2122947 | NOTCH1 Intracellular Domain Regulates Transcription | 4 | 56 | 4 | 247 | 0.00388 | 0.043 | 0.525 |
| R-HSA-72695 | Formation of the ternary complex, and subsequently, the 43S complex | 2 | 54 | 0 | 24 | 0.003741 | 0.04902 | 0.525 |
| R-HSA-918233 | TRAF3-dependent IRF activation pathway | 2 | 17 | 0 | 64 | 0.001178 | 0.051271 | 0.525 |
| R-HSA-381070 | IRE1alpha activates chaperones | 5 | 101 | 2 | 349 | 0.006998 | 0.051749 | 0.525 |

**Supplementary table 4.** Enriched REACTOME pathways for down-accumulated proteins of the 5-fluorouracil treated group

| Pathway identifier | Pathway name | #Entities found | #Entities total | #Interactors found | #Interactors total | Entities ratio | Entities pValue | Entities FDR |
| --- | --- | --- | --- | --- | --- | --- | --- | --- |
| R-HSA-5686938 | Regulation of TLR by endogenous ligand | 4 | 31 | 1 | 9 | 0.002148 | 2.19E-07 | 1.16E-04 |
| R-HSA-109606 | Intrinsic Pathway for Apoptosis | 6 | 61 | 5 | 251 | 0.004226 | 1.44E-06 | 3.81E-04 |
| R-HSA-5357769 | Caspase activation via extrinsic apoptotic signalling pathway | 5 | 32 | 4 | 175 | 0.002217 | 4.94E-06 | 8.70E-04 |
| R-HSA-140534 | Caspase activation via Death Receptors in the presence of ligand | 3 | 20 | 2 | 26 | 0.001386 | 1.40E-05 | 0.001534 |
| R-HSA-109581 | Apoptosis | 8 | 189 | 8 | 792 | 0.013095 | 1.54E-05 | 0.001534 |
| R-HSA-5357801 | Programmed Cell Death | 8 | 197 | 8 | 803 | 0.013649 | 1.74E-05 | 0.001534 |
| R-HSA-111464 | SMAC(DIABLO)-mediated dissociation of IAP:caspase complexes | 2 | 7 | 0 | 0 | 4.85E-04 | 4.04E-05 | 0.003031 |
| R-HSA-2562578 | TRIF-mediated programmed cell death | 3 | 10 | 3 | 48 | 6.93E-04 | 5.05E-05 | 0.003331 |
| R-HSA-6799990 | Metal sequestration by antimicrobial proteins | 2 | 13 | 0 | 5 | 9.01E-04 | 2.65E-04 | 0.015363 |
| R-HSA-5603041 | IRAK4 deficiency (TLR2/4) | 2 | 19 | 0 | 11 | 0.001316 | 6.36E-04 | 0.033061 |
| R-HSA-6785807 | Interleukin-4 and Interleukin-13 signaling | 4 | 211 | 1 | 139 | 0.014619 | 9.56E-04 | 0.045882 |
| R-HSA-5602498 | MyD88 deficiency (TLR2/4) | 2 | 18 | 1 | 25 | 0.001247 | 0.001223 | 0.053792 |
| R-HSA-937041 | IKK complex recruitment mediated by RIP1 | 2 | 24 | 1 | 24 | 0.001663 | 0.001481 | 0.059255 |
| R-HSA-111463 | SMAC (DIABLO) binds to IAPs | 2 | 7 | 2 | 51 | 4.85E-04 | 0.002232 | 0.079206 |
| R-HSA-111469 | SMAC, XIAP-regulated apoptotic response | 2 | 8 | 2 | 51 | 5.54E-04 | 0.002316 | 0.079206 |
| R-HSA-111459 | Activation of caspases through apoptosome-mediated cleavage | 2 | 8 | 2 | 51 | 5.54E-04 | 0.0024 | 0.079206 |
| R-HSA-168898 | Toll-like Receptor Cascades | 5 | 184 | 4 | 639 | 0.012749 | 0.002813 | 0.07933 |
| R-HSA-111453 | BH3-only proteins associate with and inactivate anti-apoptotic BCL-2 members | 2 | 11 | 2 | 58 | 7.62E-04 | 0.002938 | 0.07933 |
| R-HSA-389513 | CTLA4 inhibitory signaling | 2 | 25 | 0 | 36 | 0.001732 | 0.002938 | 0.07933 |
| R-HSA-975163 | IRAK2 mediated activation of TAK1 complex upon TLR7/8 or 9 stimulation | 2 | 18 | 0 | 69 | 0.001247 | 0.005098 | 0.125285 |
| R-HSA-937072 | TRAF6-mediated induction of TAK1 complex within TLR4 complex | 2 | 19 | 0 | 69 | 0.001316 | 0.00522 | 0.125285 |
| R-HSA-5674400 | Constitutive Signaling by AKT1 E17K in Cancer | 3 | 32 | 4 | 267 | 0.002217 | 0.006304 | 0.138691 |
| R-HSA-111461 | Cytochrome c-mediated apoptotic response | 2 | 15 | 2 | 92 | 0.001039 | 0.007506 | 0.165123 |
| R-HSA-936964 | Activation of IRF3/IRF7 mediated by TBK1/IKK epsilon | 2 | 20 | 0 | 89 | 0.001386 | 0.008402 | 0.176448 |
| R-HSA-844455 | The NLRP1 inflammasome | 1 | 5 | 1 | 4 | 3.46E-04 | 0.009148 | 0.182952 |
| R-HSA-9636249 | Inhibition of nitric oxide production | 0 | 5 | 2 | 3 | 3.46E-04 | 0.010448 | 0.192086 |
| R-HSA-264870 | Caspase-mediated cleavage of cytoskeletal proteins | 2 | 12 | 1 | 109 | 8.31E-04 | 0.010671 | 0.192086 |
| R-HSA-168643 | Nucleotide-binding domain, leucine rich repeat containing receptor (NLR) signaling pathways | 3 | 69 | 3 | 316 | 0.004781 | 0.011286 | 0.203153 |
| R-HSA-389357 | CD28 dependent PI3K/Akt signaling | 2 | 26 | 1 | 111 | 0.001801 | 0.013749 | 0.226768 |
| R-HSA-111448 | Activation of NOXA and translocation to mitochondria | 0 | 6 | 1 | 5 | 4.16E-04 | 0.014339 | 0.226768 |
| R-HSA-111471 | Apoptotic factor-mediated response | 2 | 20 | 2 | 129 | 0.001386 | 0.014523 | 0.226768 |
| R-HSA-3371378 | Regulation by c-FLIP | 1 | 11 | 2 | 3 | 7.62E-04 | 0.015632 | 0.226768 |
| R-HSA-198323 | AKT phosphorylates targets in the cytosol | 2 | 16 | 4 | 137 | 0.001109 | 0.016332 | 0.226768 |
| R-HSA-2219528 | PI3K/AKT Signaling in Cancer | 3 | 134 | 4 | 289 | 0.009284 | 0.016416 | 0.226768 |
| R-HSA-392451 | G beta:gamma signalling through PI3Kgamma | 2 | 29 | 1 | 123 | 0.002009 | 0.016747 | 0.226768 |
| R-HSA-168256 | Immune System | 13 | 2822 | 7 | 3567 | 0.195524 | 0.018218 | 0.226768 |
| R-HSA-418889 | Caspase activation via Dependence Receptors in the absence of ligand | 2 | 12 | 2 | 150 | 8.31E-04 | 0.018449 | 0.226768 |
| R-HSA-6803157 | Antimicrobial peptides | 2 | 123 | 1 | 43 | 0.008522 | 0.019106 | 0.226768 |
| R-HSA-5218900 | CASP8 activity is inhibited | 1 | 14 | 2 | 3 | 9.70E-04 | 0.019503 | 0.226768 |
| R-HSA-139915 | Activation of PUMA and translocation to mitochondria | 0 | 10 | 1 | 5 | 6.93E-04 | 0.019503 | 0.226768 |
| R-HSA-211163 | AKT-mediated inactivation of FOXO1A | 1 | 6 | 1 | 11 | 4.16E-04 | 0.02079 | 0.226768 |
| R-HSA-140342 | Apoptosis induced DNA fragmentation | 1 | 13 | 0 | 6 | 9.01E-04 | 0.022076 | 0.226768 |
| R-HSA-5260271 | Diseases of Immune System | 2 | 34 | 1 | 158 | 0.002356 | 0.023011 | 0.226768 |
| R-HSA-5602358 | Diseases associated with the TLR signaling cascade | 2 | 34 | 1 | 158 | 0.002356 | 0.023011 | 0.226768 |
| R-HSA-9007892 | Interleukin-38 signaling | 0 | 6 | 1 | 13 | 4.16E-04 | 0.024642 | 0.226768 |
| R-HSA-8941332 | RUNX2 regulates genes involved in cell migration | 1 | 14 | 0 | 5 | 9.70E-04 | 0.024642 | 0.226768 |
| R-HSA-168638 | NOD1/2 Signaling Pathway | 2 | 39 | 1 | 161 | 0.002702 | 0.024707 | 0.226768 |
| R-HSA-166166 | MyD88-independent TLR4 cascade | 3 | 107 | 3 | 427 | 0.007414 | 0.025542 | 0.226768 |
| R-HSA-937061 | TRIF(TICAM1)-mediated TLR4 signaling | 3 | 107 | 3 | 427 | 0.007414 | 0.025542 | 0.226768 |
| R-HSA-450385 | Butyrate Response Factor 1 (BRF1) binds and destabilizes mRNA | 2 | 19 | 1 | 176 | 0.001316 | 0.025949 | 0.226768 |
| R-HSA-5218920 | VEGFR2 mediated vascular permeability | 2 | 44 | 1 | 158 | 0.003049 | 0.026707 | 0.226768 |
| R-HSA-5668599 | RHO GTPases Activate NADPH Oxidases | 2 | 38 | 0 | 166 | 0.002633 | 0.029296 | 0.226768 |
| R-HSA-75108 | Activation, myristolyation of BID and translocation to mitochondria | 1 | 4 | 2 | 19 | 2.77E-04 | 0.029755 | 0.226768 |
| R-HSA-1474228 | Degradation of the extracellular matrix | 2 | 148 | 0 | 77 | 0.010254 | 0.031982 | 0.226768 |
| R-HSA-69416 | Dimerization of procaspase-8 | 1 | 11 | 1 | 25 | 7.62E-04 | 0.033572 | 0.226768 |
| R-HSA-111452 | Activation and oligomerization of BAK protein | 0 | 2 | 2 | 26 | 1.39E-04 | 0.033572 | 0.226768 |
| R-HSA-1482925 | Acyl chain remodelling of PG | 1 | 26 | 0 | 0 | 0.001801 | 0.033572 | 0.226768 |
| R-HSA-429958 | mRNA decay by 3' to 5' exoribonuclease | 1 | 24 | 0 | 4 | 0.001663 | 0.034842 | 0.226768 |
| R-HSA-211736 | Stimulation of the cell death response by PAK-2p34 | 1 | 4 | 0 | 24 | 2.77E-04 | 0.034842 | 0.226768 |
| R-HSA-9634635 | Estrogen-stimulated signaling through PRKCZ | 0 | 12 | 1 | 15 | 8.31E-04 | 0.034842 | 0.226768 |
| R-HSA-450604 | KSRP (KHSRP) binds and destabilizes mRNA | 2 | 20 | 1 | 207 | 0.001386 | 0.035045 | 0.226768 |
| R-HSA-5660668 | CLEC7A/inflammasome pathway | 1 | 8 | 1 | 25 | 5.54E-04 | 0.03864 | 0.226768 |
| R-HSA-205025 | NADE modulates death signalling | 1 | 6 | 0 | 25 | 4.16E-04 | 0.03864 | 0.226768 |
| R-HSA-1482922 | Acyl chain remodelling of PI | 1 | 25 | 0 | 5 | 0.001732 | 0.03864 | 0.226768 |
| R-HSA-174490 | Membrane binding and targetting of GAG proteins | 0 | 17 | 1 | 14 | 0.001178 | 0.039903 | 0.226768 |
| R-HSA-174495 | Synthesis And Processing Of GAG, GAGPOL Polyproteins | 0 | 18 | 1 | 14 | 0.001247 | 0.041164 | 0.226768 |
| R-HSA-397795 | G-protein beta:gamma signalling | 2 | 39 | 1 | 213 | 0.002702 | 0.041499 | 0.226768 |
| R-HSA-139910 | Activation of BMF and translocation to mitochondria | 0 | 5 | 2 | 29 | 3.46E-04 | 0.042424 | 0.226768 |
| R-HSA-114294 | Activation, translocation and oligomerization of BAX | 0 | 2 | 2 | 33 | 1.39E-04 | 0.042424 | 0.226768 |
| R-HSA-376172 | DSCAM interactions | 0 | 15 | 2 | 21 | 0.001039 | 0.043682 | 0.226768 |
| R-HSA-6803211 | TP53 Regulates Transcription of Death Receptors and Ligands | 0 | 18 | 1 | 19 | 0.001247 | 0.043682 | 0.226768 |
| R-HSA-1482801 | Acyl chain remodelling of PS | 1 | 31 | 0 | 3 | 0.002148 | 0.043682 | 0.226768 |
| R-HSA-5674404 | PTEN Loss of Function in Cancer | 0 | 3 | 1 | 32 | 2.08E-04 | 0.044939 | 0.226768 |
| R-HSA-111447 | Activation of BAD and translocation to mitochondria | 1 | 19 | 2 | 26 | 0.001316 | 0.047447 | 0.226768 |
| R-HSA-5675482 | Regulation of necroptotic cell death | 1 | 21 | 2 | 24 | 0.001455 | 0.047447 | 0.226768 |
| R-HSA-111446 | Activation of BIM and translocation to mitochondria | 0 | 5 | 2 | 34 | 3.46E-04 | 0.048699 | 0.226768 |
| R-HSA-168927 | TICAM1, RIP1-mediated IKK complex recruitment | 0 | 19 | 1 | 24 | 0.001316 | 0.048699 | 0.226768 |
| R-HSA-389356 | CD28 co-stimulation | 2 | 39 | 1 | 238 | 0.002702 | 0.049011 | 0.226768 |
| R-HSA-1236974 | ER-Phagosome pathway | 2 | 165 | 0 | 115 | 0.011432 | 0.049334 | 0.226768 |
| R-HSA-9603505 | NTRK3 as a dependence receptor | 0 | 3 | 2 | 37 | 2.08E-04 | 0.049949 | 0.226768 |
| R-HSA-1810476 | RIP-mediated NFkB activation via ZBP1 | 0 | 19 | 1 | 24 | 0.001316 | 0.051198 | 0.226768 |

**Supplementary table 5.** Enriched REACTOME pathways for up-accumulated proteins of the group treated with Agarikon.1 and 5-fluorouracil

| Pathway identifier | Pathway name | #Entities found | #Entities total | #Interactors found | #Interactors total | Entities ratio | Entities pValue | Entities FDR |
| --- | --- | --- | --- | --- | --- | --- | --- | --- |
| R-HSA-8957275 | Post-translational protein phosphorylation | 6 | 109 | 0 | 0 | 0.007650197 | 2.52E-06 | 0.00164 |
| R-HSA-390522 | Striated Muscle Contraction | 4 | 40 | 0 | 3 | 0.002807412 | 1.74E-05 | 0.00564 |
| R-HSA-8963888 | Chylomicron assembly | 4 | 14 | 2 | 36 | 9.83E-04 | 2.66E-05 | 0.00577 |
| R-HSA-1989781 | PPARA activates gene expression | 9 | 174 | 3 | 342 | 0.01221224 | 6.66E-05 | 0.0108 |
| R-HSA-400206 | Regulation of lipid metabolism by Peroxisome proliferator-activated receptor alpha (PPARalpha) | 9 | 176 | 3 | 372 | 0.012352611 | 9.75E-05 | 0.01267 |
| R-HSA-381426 | Regulation of Insulin-like Growth Factor (IGF) transport and uptake by Insulin-like Growth Factor Binding Proteins (IGFBPs) | 6 | 127 | 0 | 103 | 0.008913532 | 1.49E-04 | 0.0161 |
| R-HSA-8963898 | Plasma lipoprotein assembly | 4 | 30 | 2 | 74 | 0.002105559 | 4.71E-04 | 0.03679 |
| R-HSA-381119 | Unfolded Protein Response (UPR) | 10 | 155 | 4 | 681 | 0.01087872 | 5.41E-04 | 0.03679 |
| R-HSA-381183 | ATF6 (ATF6-alpha) activates chaperone genes | 4 | 15 | 3 | 98 | 0.001052779 | 5.82E-04 | 0.03679 |
| R-HSA-8963901 | Chylomicron remodeling | 3 | 17 | 0 | 30 | 0.00119315 | 5.90E-04 | 0.03679 |
| R-HSA-381033 | ATF6 (ATF6-alpha) activates chaperones | 4 | 17 | 3 | 98 | 0.00119315 | 6.23E-04 | 0.03679 |
| R-HSA-381070 | IRE1alpha activates chaperones | 7 | 101 | 1 | 347 | 0.007088714 | 9.16E-04 | 0.04946 |
| R-HSA-8963899 | Plasma lipoprotein remodeling | 4 | 54 | 1 | 81 | 0.003790006 | 0.001187 | 0.05936 |
| R-HSA-174824 | Plasma lipoprotein assembly, remodeling, and clearance | 5 | 98 | 2 | 178 | 0.006878158 | 0.0024 | 0.11039 |
| R-HSA-381038 | XBP1(S) activates chaperone genes | 6 | 95 | 1 | 343 | 0.006667602 | 0.004238 | 0.18223 |
| R-HSA-975634 | Retinoid metabolism and transport | 3 | 79 | 0 | 33 | 0.005544638 | 0.006381 | 0.24247 |
| R-HSA-397014 | Muscle contraction | 5 | 256 | 1 | 125 | 0.017967434 | 0.009835 | 0.35405 |
| R-HSA-3000480 | Scavenging by Class A Receptors | 2 | 48 | 0 | 0 | 0.003368894 | 0.012432 | 0.42267 |
| R-HSA-8963889 | Assembly of active LPL and LIPC lipase complexes | 2 | 30 | 0 | 37 | 0.002105559 | 0.023216 | 0.52487 |
| R-HSA-5682113 | Defective ABCA1 causes Tangier disease | 1 | 5 | 0 | 2 | 3.51E-04 | 0.024151 | 0.52487 |
| R-HSA-381340 | Transcriptional regulation of white adipocyte differentiation | 5 | 108 | 1 | 400 | 0.007580011 | 0.027044 | 0.52487 |
| R-HSA-6796648 | TP53 Regulates Transcription of DNA Repair Genes | 3 | 89 | 2 | 113 | 0.006246491 | 0.030712 | 0.52487 |
| R-HSA-6806667 | Metabolism of fat-soluble vitamins | 3 | 94 | 0 | 112 | 0.006597417 | 0.033162 | 0.52487 |
| R-HSA-556833 | Metabolism of lipids | 16 | 1445 | 7 | 1531 | 0.101417743 | 0.037035 | 0.52487 |
| R-HSA-611105 | Respiratory electron transport | 3 | 115 | 1 | 148 | 0.008071308 | 0.041572 | 0.52487 |
| R-HSA-1428517 | The citric acid (TCA) cycle and respiratory electron transport | 4 | 229 | 1 | 208 | 0.016072431 | 0.047433 | 0.52487 |
| R-HSA-70263 | Gluconeogenesis | 2 | 66 | 0 | 44 | 0.004632229 | 0.055094 | 0.52487 |
| R-HSA-163200 | Respiratory electron transport, ATP synthesis by chemiosmotic coupling, and heat production by uncoupling proteins. | 3 | 146 | 1 | 148 | 0.010247052 | 0.057365 | 0.52487 |

**Supplementary table 6.** Enriched REACTOME pathways for down-accumulated proteins of the group treated with Agarikon.1 and 5-fluorouracil

| Pathway identifier | Pathway name | #Entities found | #Entities total | #Interactors found | #Interactors total | Entities ratio | Entities pValue | Entities FDR |
| --- | --- | --- | --- | --- | --- | --- | --- | --- |
| R-HSA-156902 | Peptide chain elongation | 17 | 97 | 1 | 28 | 0.006808 | 1.11E-16 | 1.34E-14 |
| R-HSA-72689 | Formation of a pool of free 40S subunits | 18 | 106 | 0 | 6 | 0.00744 | 1.11E-16 | 1.34E-14 |
| R-HSA-975956 | Nonsense Mediated Decay (NMD) independent of the Exon Junction Complex (EJC) | 17 | 101 | 0 | 23 | 0.007089 | 1.11E-16 | 1.34E-14 |
| R-HSA-72706 | GTP hydrolysis and joining of the 60S ribosomal subunit | 20 | 120 | 4 | 47 | 0.008422 | 1.11E-16 | 1.34E-14 |
| R-HSA-156827 | L13a-mediated translational silencing of Ceruloplasmin expression | 20 | 120 | 0 | 2 | 0.008422 | 1.11E-16 | 1.34E-14 |
| R-HSA-1799339 | SRP-dependent cotranslational protein targeting to membrane | 17 | 119 | 0 | 0 | 0.008352 | 1.11E-16 | 1.34E-14 |
| R-HSA-72764 | Eukaryotic Translation Termination | 17 | 106 | 0 | 6 | 0.00744 | 1.11E-16 | 1.34E-14 |
| R-HSA-2408557 | Selenocysteine synthesis | 17 | 113 | 0 | 2 | 0.007931 | 1.11E-16 | 1.34E-14 |
| R-HSA-9010553 | Regulation of expression of SLITs and ROBOs | 22 | 183 | 5 | 84 | 0.012844 | 1.11E-16 | 1.34E-14 |
| R-HSA-2408522 | Selenoamino acid metabolism | 20 | 181 | 0 | 15 | 0.012704 | 1.11E-16 | 1.34E-14 |
| R-HSA-192823 | Viral mRNA Translation | 17 | 114 | 0 | 22 | 0.008001 | 4.44E-16 | 4.88E-14 |
| R-HSA-72613 | Eukaryotic Translation Initiation | 20 | 130 | 5 | 107 | 0.009124 | 5.55E-16 | 5.16E-14 |
| R-HSA-72737 | Cap-dependent Translation Initiation | 20 | 130 | 5 | 106 | 0.009124 | 5.55E-16 | 5.16E-14 |
| R-HSA-72702 | Ribosomal scanning and start codon recognition | 13 | 64 | 0 | 8 | 0.004492 | 7.88E-15 | 6.78E-13 |
| R-HSA-156842 | Eukaryotic Translation Elongation | 17 | 102 | 2 | 66 | 0.007159 | 9.21E-15 | 7.37E-13 |
| R-HSA-927802 | Nonsense-Mediated Decay (NMD) | 17 | 124 | 4 | 77 | 0.008703 | 7.51E-14 | 5.33E-12 |
| R-HSA-975957 | Nonsense Mediated Decay (NMD) enhanced by the Exon Junction Complex (EJC) | 17 | 124 | 4 | 77 | 0.008703 | 7.51E-14 | 5.33E-12 |
| R-HSA-72649 | Translation initiation complex formation | 13 | 62 | 1 | 32 | 0.004351 | 2.61E-13 | 1.75E-11 |
| R-HSA-72766 | Translation | 27 | 339 | 6 | 344 | 0.023793 | 5.79E-13 | 3.65E-11 |
| R-HSA-168273 | Influenza Viral RNA Transcription and Replication | 18 | 175 | 1 | 88 | 0.012282 | 8.56E-13 | 5.13E-11 |
| R-HSA-376176 | Signaling by ROBO receptors | 24 | 235 | 8 | 325 | 0.016494 | 2.21E-12 | 1.26E-10 |
| R-HSA-72695 | Formation of the ternary complex, and subsequently, the 43S complex | 11 | 54 | 1 | 24 | 0.00379 | 2.42E-11 | 1.33E-09 |
| R-HSA-72662 | Activation of the mRNA upon binding of the cap-binding complex and eIFs, and subsequent binding to 43S | 13 | 66 | 4 | 76 | 0.004632 | 2.91E-11 | 1.51E-09 |
| R-HSA-168255 | Influenza Life Cycle | 18 | 188 | 1 | 224 | 0.013195 | 9.95E-10 | 4.98E-08 |
| R-HSA-168254 | Influenza Infection | 18 | 199 | 1 | 264 | 0.013967 | 5.29E-09 | 2.54E-07 |
| R-HSA-71291 | Metabolism of amino acids and derivatives | 25 | 660 | 2 | 273 | 0.046322 | 1.74E-08 | 8.02E-07 |
| R-HSA-8950505 | Gene and protein expression by JAK-STAT signaling after Interleukin-12 stimulation | 16 | 73 | 9 | 325 | 0.005124 | 4.19E-08 | 1.84E-06 |
| R-HSA-379716 | Cytosolic tRNA aminoacylation | 7 | 50 | 0 | 10 | 0.003509 | 3.55E-07 | 1.52E-05 |
| R-HSA-9020591 | Interleukin-12 signaling | 16 | 84 | 11 | 391 | 0.005896 | 3.88E-07 | 1.59E-05 |
| R-HSA-6791226 | Major pathway of rRNA processing in the nucleolus and cytosol | 17 | 189 | 3 | 377 | 0.013265 | 4.86E-07 | 1.94E-05 |
| R-HSA-8868773 | rRNA processing in the nucleus and cytosol | 17 | 207 | 3 | 382 | 0.014528 | 8.44E-07 | 3.29E-05 |
| R-HSA-8953854 | Metabolism of RNA | 37 | 782 | 26 | 1643 | 0.054885 | 1.03E-06 | 3.80E-05 |
| R-HSA-72312 | rRNA processing | 17 | 245 | 3 | 425 | 0.017195 | 5.09E-06 | 1.83E-04 |
| R-HSA-447115 | Interleukin-12 family signaling | 16 | 96 | 14 | 569 | 0.006738 | 2.71E-05 | 9.49E-04 |
| R-HSA-73863 | RNA Polymerase I Transcription Termination | 5 | 33 | 2 | 21 | 0.002316 | 5.20E-05 | 0.001716347 |
| R-HSA-1236978 | Cross-presentation of soluble exogenous antigens (endosomes) | 5 | 53 | 0 | 0 | 0.00372 | 5.20E-05 | 0.001716347 |
| R-HSA-174113 | SCF-beta-TrCP mediated degradation of Emi1 | 5 | 55 | 0 | 0 | 0.00386 | 6.19E-05 | 0.001979478 |
| R-HSA-350562 | Regulation of ornithine decarboxylase (ODC) | 5 | 51 | 0 | 10 | 0.003579 | 7.31E-05 | 0.002265689 |
| R-HSA-180585 | Vif-mediated degradation of APOBEC3G | 5 | 56 | 0 | 2 | 0.00393 | 7.31E-05 | 0.002265689 |
| R-HSA-174084 | Autodegradation of Cdh1 by Cdh1:APC/C | 5 | 63 | 0 | 0 | 0.004422 | 1.16E-04 | 0.003374466 |
| R-HSA-5362768 | Hh mutants that don't undergo autocatalytic processing are degraded by ERAD | 5 | 61 | 0 | 2 | 0.004281 | 1.16E-04 | 0.003374466 |
| R-HSA-68827 | CDT1 association with the CDC6:ORC:origin complex | 5 | 59 | 1 | 9 | 0.004141 | 1.44E-04 | 0.004039309 |
| R-HSA-5387390 | Hh mutants abrogate ligand secretion | 5 | 64 | 0 | 3 | 0.004492 | 1.55E-04 | 0.004329161 |
| R-HSA-4641257 | Degradation of AXIN | 5 | 57 | 0 | 16 | 0.004001 | 1.89E-04 | 0.005105698 |
| R-HSA-174154 | APC/C:Cdc20 mediated degradation of Securin | 5 | 67 | 0 | 4 | 0.004702 | 2.02E-04 | 0.005246989 |
| R-HSA-174178 | APC/C:Cdh1 mediated degradation of Cdc20 and other APC/C:Cdh1 targeted proteins in late mitosis/early G1 | 5 | 72 | 0 | 0 | 0.005053 | 2.15E-04 | 0.005499959 |
| R-HSA-211733 | Regulation of activated PAK-2p34 by proteasome mediated degradation | 5 | 50 | 1 | 24 | 0.003509 | 2.29E-04 | 0.005499959 |
| R-HSA-5658442 | Regulation of RAS by GAPs | 5 | 71 | 0 | 2 | 0.004983 | 2.29E-04 | 0.005499959 |
| R-HSA-5663205 | Infectious disease | 25 | 540 | 11 | 1148 | 0.0379 | 2.54E-04 | 0.006085407 |
| R-HSA-68949 | Orc1 removal from chromatin | 5 | 73 | 0 | 10 | 0.005124 | 2.92E-04 | 0.006721988 |
| R-HSA-5676590 | NIK-->noncanonical NF-kB signaling | 5 | 61 | 1 | 22 | 0.004281 | 3.28E-04 | 0.007223835 |
| R-HSA-69017 | CDK-mediated phosphorylation and removal of Cdc6 | 5 | 74 | 0 | 5 | 0.005194 | 3.28E-04 | 0.007223835 |
| R-HSA-169911 | Regulation of Apoptosis | 5 | 54 | 2 | 31 | 0.00379 | 4.33E-04 | 0.00866715 |
| R-HSA-5607761 | Dectin-1 mediated noncanonical NF-kB signaling | 5 | 66 | 1 | 22 | 0.004632 | 4.33E-04 | 0.00866715 |
| R-HSA-349425 | Autodegradation of the E3 ubiquitin ligase COP1 | 5 | 54 | 0 | 31 | 0.00379 | 4.33E-04 | 0.00866715 |
| R-HSA-5678895 | Defective CFTR causes cystic fibrosis | 5 | 67 | 0 | 19 | 0.004702 | 4.33E-04 | 0.00866715 |
| R-HSA-68867 | Assembly of the pre-replicative complex | 5 | 68 | 1 | 30 | 0.004773 | 5.34E-04 | 0.010682978 |
| R-HSA-69610 | p53-Independent DNA Damage Response | 5 | 54 | 1 | 41 | 0.00379 | 6.84E-04 | 0.012717009 |
| R-HSA-69613 | p53-Independent G1/S DNA damage checkpoint | 5 | 54 | 1 | 41 | 0.00379 | 6.84E-04 | 0.012717009 |
| R-HSA-69601 | Ubiquitin Mediated Degradation of Phosphorylated Cdc25A | 5 | 54 | 1 | 41 | 0.00379 | 6.84E-04 | 0.012717009 |
| R-HSA-450408 | AUF1 (hnRNP D0) binds and destabilizes mRNA | 7 | 56 | 5 | 147 | 0.00393 | 7.07E-04 | 0.012717009 |
| R-HSA-1168372 | Downstream signaling events of B Cell Receptor (BCR) | 6 | 93 | 1 | 61 | 0.006527 | 8.13E-04 | 0.01462538 |
| R-HSA-174184 | Cdc20:Phospho-APC/C mediated degradation of Cyclin A | 5 | 72 | 0 | 26 | 0.005053 | 8.24E-04 | 0.014840575 |
| R-HSA-1234174 | Cellular response to hypoxia | 7 | 85 | 2 | 135 | 0.005966 | 8.65E-04 | 0.015340987 |
| R-HSA-1169091 | Activation of NF-kappaB in B cells | 5 | 72 | 0 | 33 | 0.005053 | 9.02E-04 | 0.015340987 |
| R-HSA-6798695 | Neutrophil degranulation | 11 | 480 | 0 | 0 | 0.033689 | 9.37E-04 | 0.015934399 |
| R-HSA-4608870 | Asymmetric localization of PCP proteins | 5 | 66 | 0 | 38 | 0.004632 | 0.001121 | 0.019060008 |
| R-HSA-179419 | APC:Cdc20 mediated degradation of cell cycle proteins prior to satisfation of the cell cycle checkpoint | 5 | 73 | 0 | 37 | 0.005124 | 0.001219 | 0.020222338 |
| R-HSA-379724 | tRNA Aminoacylation | 7 | 68 | 0 | 152 | 0.004773 | 0.001264 | 0.020222338 |
| R-HSA-1234176 | Oxygen-dependent proline hydroxylation of Hypoxia-inducible Factor Alpha | 5 | 72 | 1 | 47 | 0.005053 | 0.001433 | 0.022926556 |
| R-HSA-176409 | APC/C:Cdc20 mediated degradation of mitotic proteins | 5 | 75 | 0 | 41 | 0.005264 | 0.00149 | 0.023847875 |
| R-HSA-75815 | Ubiquitin-dependent degradation of Cyclin D | 5 | 54 | 1 | 61 | 0.00379 | 0.001673 | 0.026775627 |
| R-HSA-5358346 | Hedgehog ligand biogenesis | 5 | 72 | 0 | 44 | 0.005053 | 0.001804 | 0.027064438 |
| R-HSA-69052 | Switching of origins to a post-replicative state | 5 | 92 | 0 | 39 | 0.006457 | 0.001942 | 0.029136886 |
| R-HSA-5619084 | ABC transporter disorders | 5 | 99 | 0 | 21 | 0.006948 | 0.001942 | 0.029136886 |
| R-HSA-5610780 | Degradation of GLI1 by the proteasome | 5 | 62 | 0 | 62 | 0.004351 | 0.002322 | 0.034426704 |
| R-HSA-69239 | Synthesis of DNA | 6 | 132 | 1 | 70 | 0.009264 | 0.002459 | 0.034426704 |
| R-HSA-69002 | DNA Replication Pre-Initiation | 5 | 88 | 1 | 54 | 0.006176 | 0.002752 | 0.038526927 |
| R-HSA-9604323 | Negative regulation of NOTCH4 signaling | 5 | 57 | 0 | 76 | 0.004001 | 0.003036 | 0.042508172 |
| R-HSA-187577 | SCF(Skp2)-mediated degradation of p27/p21 | 5 | 62 | 0 | 79 | 0.004351 | 0.003341 | 0.043657087 |
| R-HSA-69481 | G2/M Checkpoints | 6 | 154 | 1 | 56 | 0.010809 | 0.003358 | 0.043657087 |
| R-HSA-351202 | Metabolism of polyamines | 5 | 80 | 0 | 63 | 0.005615 | 0.003448 | 0.044820273 |
| R-HSA-422475 | Axon guidance | 25 | 584 | 22 | 1474 | 0.040988 | 0.004086 | 0.053113978 |
| R-HSA-5668541 | TNFR2 non-canonical NF-kB pathway | 6 | 104 | 1 | 121 | 0.007299 | 0.004482 | 0.058271378 |
| R-HSA-4641258 | Degradation of DVL | 5 | 57 | 0 | 93 | 0.004001 | 0.004518 | 0.058730003 |
| R-HSA-2467813 | Separation of Sister Chromatids | 6 | 194 | 0 | 27 | 0.013616 | 0.004694 | 0.061019842 |
| R-HSA-5610783 | Degradation of GLI2 by the proteasome | 5 | 62 | 3 | 88 | 0.004351 | 0.005205 | 0.063053621 |
| R-HSA-69306 | DNA Replication | 6 | 141 | 1 | 98 | 0.009896 | 0.005254 | 0.063053621 |
| R-HSA-5610785 | GLI3 is processed to GLI3R by the proteasome | 5 | 62 | 3 | 91 | 0.004351 | 0.005351 | 0.064207945 |
| R-HSA-5689880 | Ub-specific processing proteases | 8 | 206 | 1 | 172 | 0.014458 | 0.005806 | 0.069675136 |
| R-HSA-68882 | Mitotic Anaphase | 6 | 208 | 0 | 30 | 0.014599 | 0.006521 | 0.078251585 |
| R-HSA-140342 | Apoptosis induced DNA fragmentation | 2 | 13 | 0 | 6 | 9.12E-04 | 0.007196 | 0.08635193 |
| R-HSA-176814 | Activation of APC/C and APC/C:Cdc20 mediated degradation of mitotic proteins | 5 | 76 | 0 | 92 | 0.005334 | 0.007333 | 0.08799299 |
| R-HSA-382556 | ABC-family proteins mediated transport | 5 | 124 | 0 | 44 | 0.008703 | 0.007518 | 0.090217799 |
| R-HSA-5689603 | UCH proteinases | 5 | 98 | 7 | 121 | 0.006878 | 0.0087 | 0.095696407 |
| R-HSA-69541 | Stabilization of p53 | 5 | 59 | 2 | 117 | 0.004141 | 0.0087 | 0.095696407 |
| R-HSA-69656 | Cyclin A:Cdk2-associated events at S phase entry | 6 | 90 | 3 | 177 | 0.006317 | 0.010446 | 0.114907927 |
| R-HSA-8854050 | FBXL7 down-regulates AURKA during mitotic entry and in early mitosis | 5 | 55 | 2 | 127 | 0.00386 | 0.010945 | 0.120389942 |
| R-HSA-176408 | Regulation of APC/C activators between G1/S and early anaphase | 5 | 82 | 0 | 115 | 0.005755 | 0.011189 | 0.123076839 |
| R-HSA-73772 | RNA Polymerase I Promoter Escape | 3 | 64 | 0 | 0 | 0.004492 | 0.012196 | 0.13415662 |
| R-HSA-8941858 | Regulation of RUNX3 expression and activity | 5 | 57 | 1 | 131 | 0.004001 | 0.012466 | 0.137127013 |
| R-HSA-8852276 | The role of GTSE1 in G2/M progression after G2 checkpoint | 5 | 83 | 2 | 110 | 0.005825 | 0.013004 | 0.143039443 |
| R-HSA-449147 | Signaling by Interleukins | 26 | 639 | 25 | 1811 | 0.044848 | 0.01438 | 0.158178163 |
| R-HSA-5687128 | MAPK6/MAPK4 signaling | 8 | 106 | 5 | 343 | 0.00744 | 0.014987 | 0.16485388 |
| R-HSA-69206 | G1/S Transition | 8 | 150 | 3 | 339 | 0.010528 | 0.018006 | 0.180061342 |
| R-HSA-2555396 | Mitotic Metaphase and Anaphase | 6 | 211 | 0 | 87 | 0.014809 | 0.018248 | 0.182475666 |
| R-HSA-73980 | RNA Polymerase III Transcription Termination | 2 | 23 | 0 | 5 | 0.001614 | 0.018519 | 0.185194688 |
| R-HSA-194313 | VEGF ligand-receptor interactions | 2 | 8 | 4 | 25 | 5.61E-04 | 0.018519 | 0.185194688 |
| R-HSA-195399 | VEGF binds to VEGFR leading to receptor dimerization | 2 | 8 | 4 | 25 | 5.61E-04 | 0.018519 | 0.185194688 |
| R-HSA-180534 | Vpu mediated degradation of CD4 | 5 | 53 | 1 | 158 | 0.00372 | 0.018907 | 0.189071189 |
| R-HSA-69202 | Cyclin E associated events during G1/S transition | 6 | 88 | 3 | 227 | 0.006176 | 0.021554 | 0.215540844 |
| R-HSA-174143 | APC/C-mediated degradation of cell cycle proteins | 5 | 90 | 0 | 147 | 0.006317 | 0.022625 | 0.226251373 |
| R-HSA-453276 | Regulation of mitotic cell cycle | 5 | 90 | 0 | 147 | 0.006317 | 0.022625 | 0.226251373 |
| R-HSA-69563 | p53-Dependent G1 DNA Damage Response | 5 | 70 | 4 | 167 | 0.004913 | 0.025919 | 0.257235624 |
| R-HSA-69580 | p53-Dependent G1/S DNA damage checkpoint | 5 | 70 | 4 | 167 | 0.004913 | 0.025919 | 0.257235624 |
| R-HSA-8939236 | RUNX1 regulates transcription of genes involved in differentiation of HSCs | 5 | 106 | 1 | 128 | 0.00744 | 0.028582 | 0.257235624 |
| R-HSA-8939902 | Regulation of RUNX2 expression and activity | 5 | 83 | 1 | 154 | 0.005825 | 0.03045 | 0.27404696 |
| R-HSA-69615 | G1/S DNA Damage Checkpoints | 5 | 72 | 5 | 181 | 0.005053 | 0.032891 | 0.296014843 |
| R-HSA-2871837 | FCERI mediated NF-kB activation | 5 | 175 | 2 | 93 | 0.012282 | 0.037044 | 0.333395557 |
| R-HSA-114608 | Platelet degranulation | 4 | 137 | 0 | 32 | 0.009615 | 0.037287 | 0.335580674 |
| R-HSA-9020702 | Interleukin-1 signaling | 6 | 109 | 4 | 267 | 0.00765 | 0.038776 | 0.348982342 |
| R-HSA-69242 | S Phase | 7 | 179 | 4 | 297 | 0.012563 | 0.041591 | 0.374322364 |
| R-HSA-9013694 | Signaling by NOTCH4 | 5 | 92 | 0 | 176 | 0.006457 | 0.043855 | 0.394694238 |
| R-HSA-5619115 | Disorders of transmembrane transporters | 6 | 282 | 1 | 68 | 0.019792 | 0.045171 | 0.406538047 |
| R-HSA-450531 | Regulation of mRNA stability by proteins that bind AU-rich elements | 8 | 93 | 11 | 458 | 0.006527 | 0.045712 | 0.411412123 |
| R-HSA-75205 | Dissolution of Fibrin Clot | 2 | 14 | 0 | 34 | 9.83E-04 | 0.049497 | 0.440746645 |
| R-HSA-5632928 | Defective Mismatch Repair Associated With MSH2 | 0 | 3 | 2 | 5 | 2.11E-04 | 0.050338 | 0.440746645 |
