# Supplementary material for: Treatment With Medicinal Mushroom Extract Mixture Inhibits Translation and Reprograms Metabolism in Advanced Colorectal Cancer Animal Model as Evidenced by Tandem Mass Tags Proteomics Analysis
Source: Front Pharmacol. 2020 Aug 21;11:1202. doi: 10.3389/fphar.2020.01202 (PMC7472604; doi:10.3389/fphar.2020.01202)

| 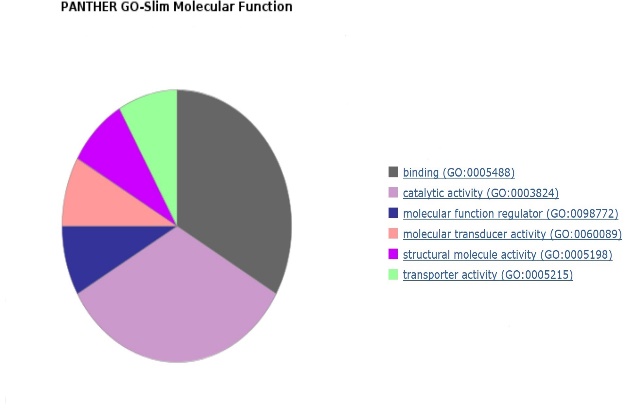 | 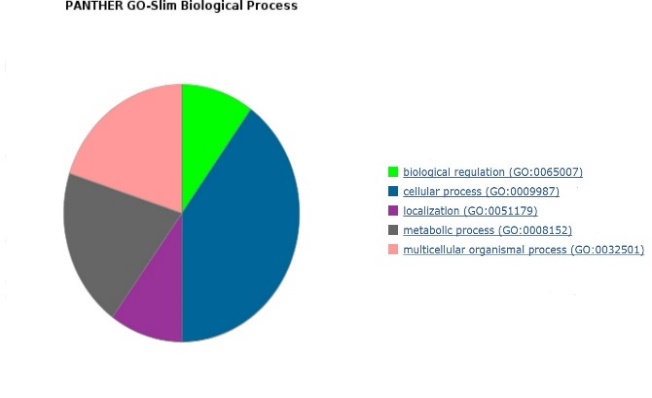 |
| --- | --- |
| (**a**) | |
| 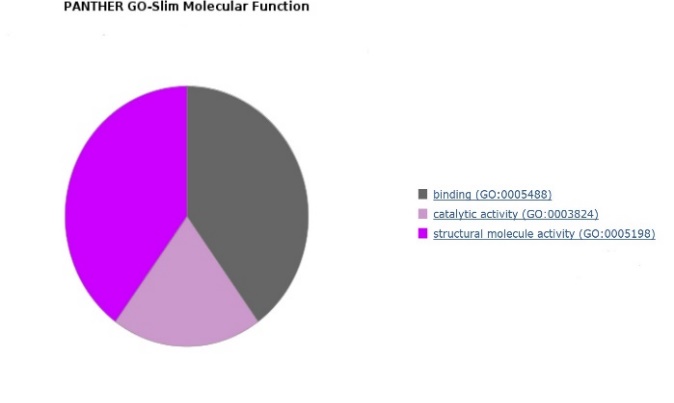 | 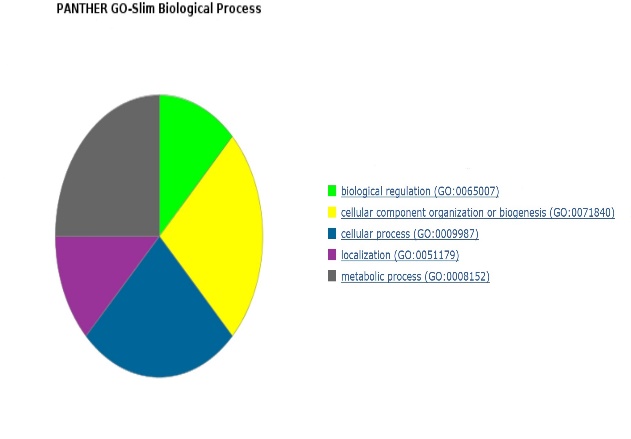 |
| (**b**) | |
| 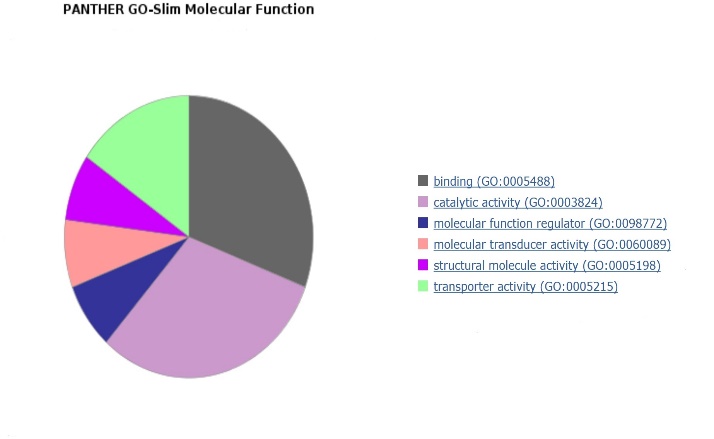 | 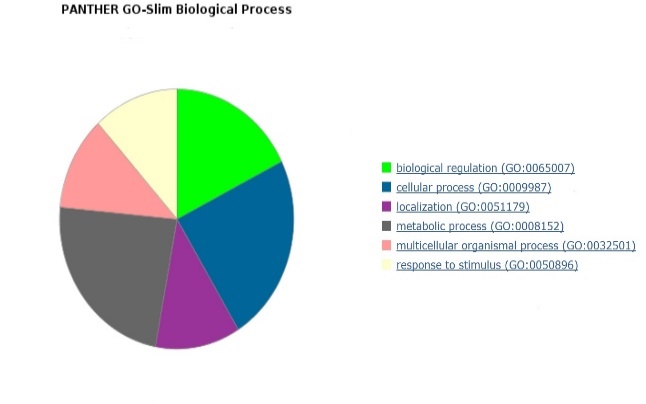 |
| (**c**) | |
| 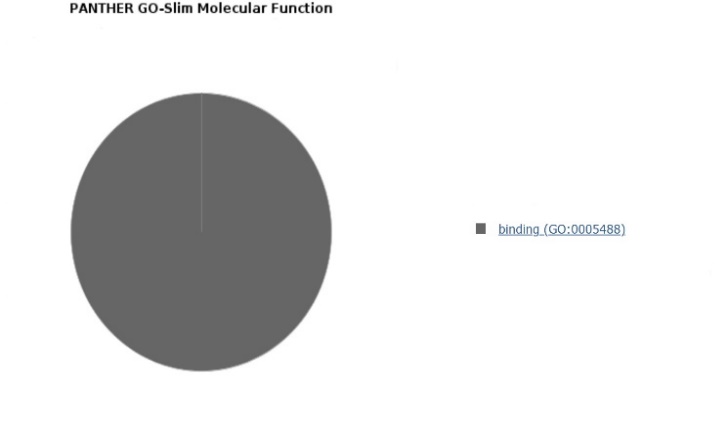 | 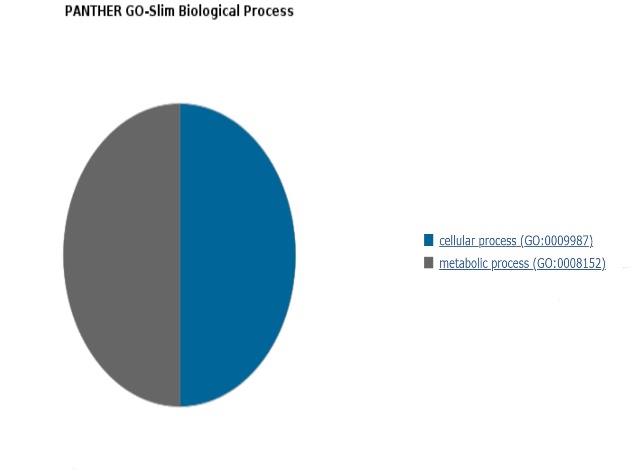 |
| (**d**) | |
| 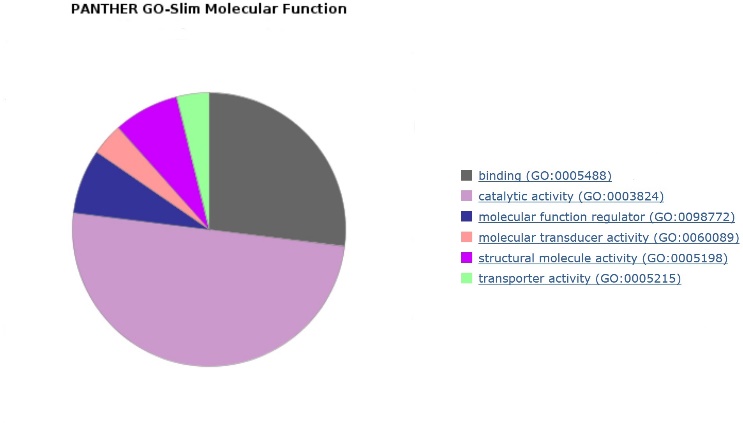 | 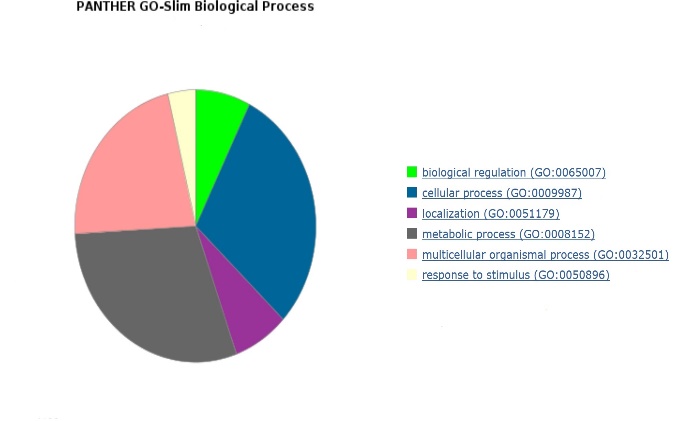 |
| (**e**) | |
| 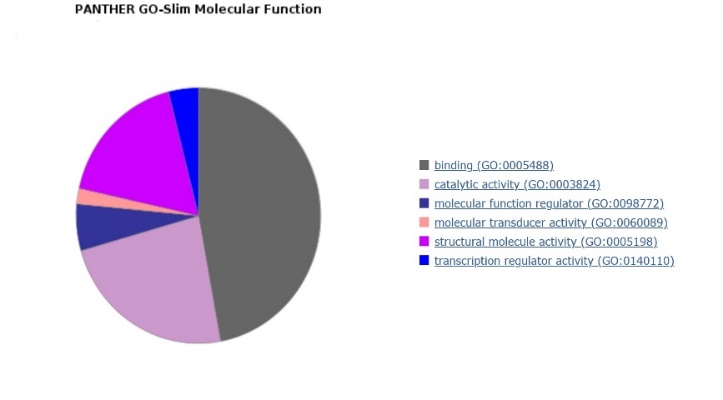 | 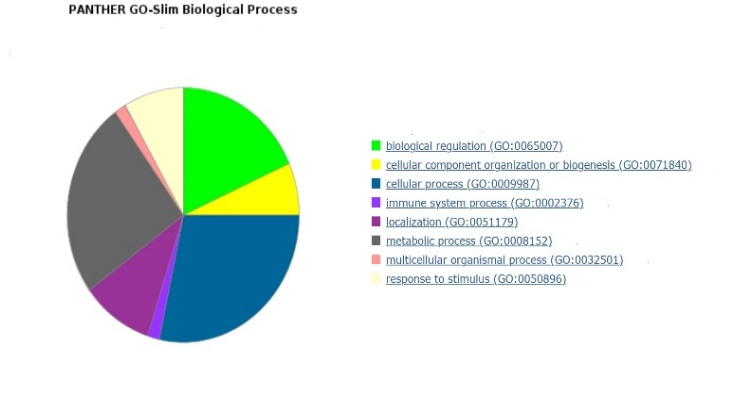 |
| (**f**) | |
| **Supplementary figure 1**. GO analysis of molecular function and biological process of the up- and down-accumulated proteins in treatment groups; (a) Agarikon.1 group up-accumulated proteins, (b) Agarikon.1 group down-accumulated proteins, (c) 5-fluorouracil group up-accumulated proteins, (d) 5-fluorouracil group down-accumulated proteins, (e) combinatorial group (AG.1 with 5-FU) up-accumulated proteins, (f) combinatorial group (AG.1 with 5-FU) down-accumulated proteins. | |


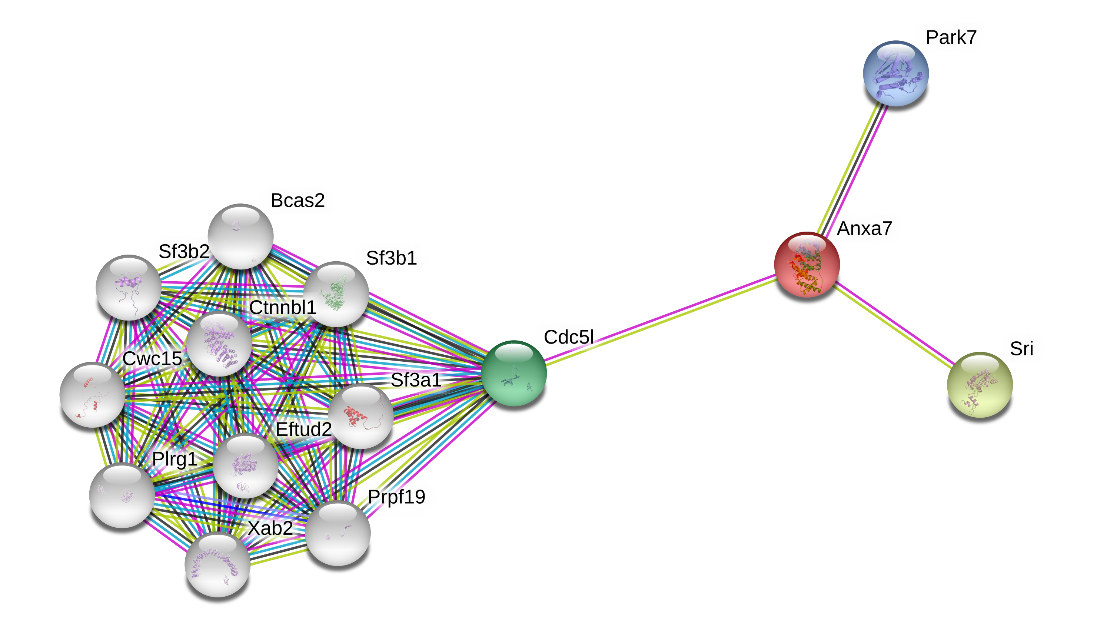


**Supplementary figure 2.** STRING enriched analysis of Anxa 7 and Cdc5l protein interactions in the Agarikon.1 down-accumulated group.


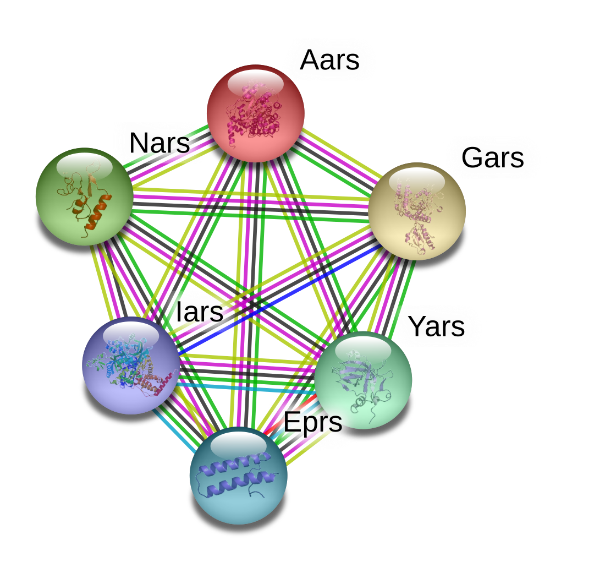


**Supplementary figure 3.** STRING enriched analysis of Aars protein interactions in the Agarikon.1 and 5-fluorouracil down-accumulated group.


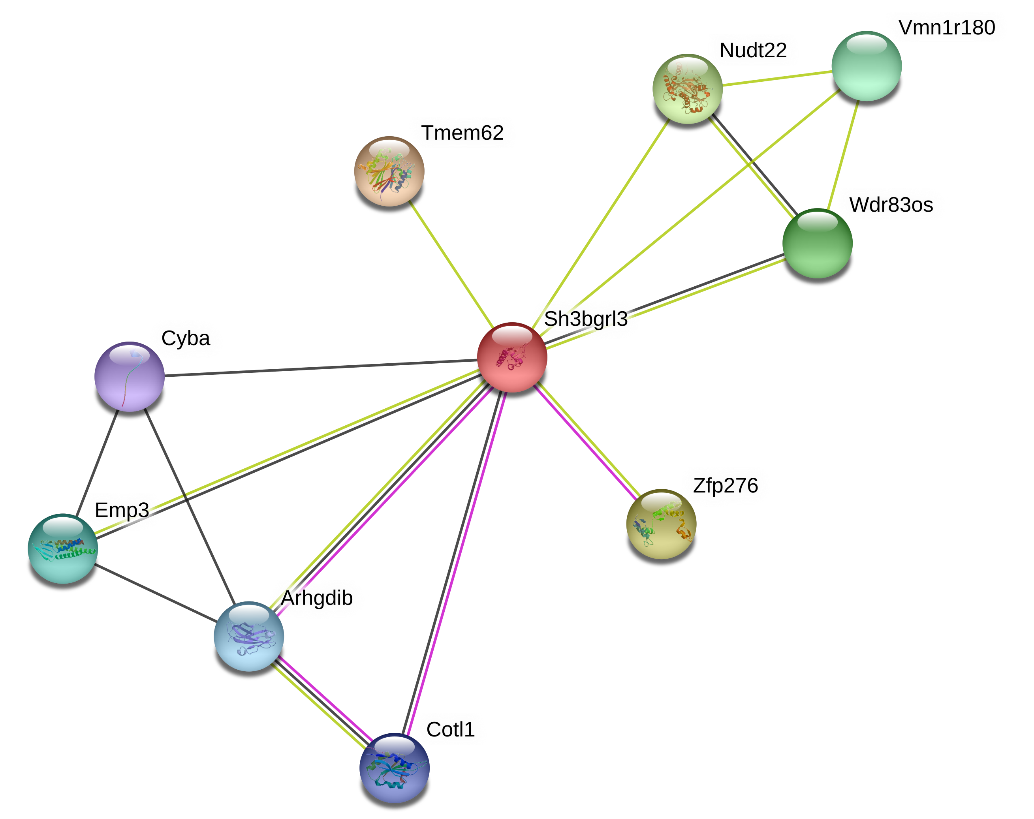


**Supplementary figure 4.** STRING enriched analysis of Sh3bgrl3 protein interactions in the Agarikon.1 and 5-fluorouracil down-accumulated group.


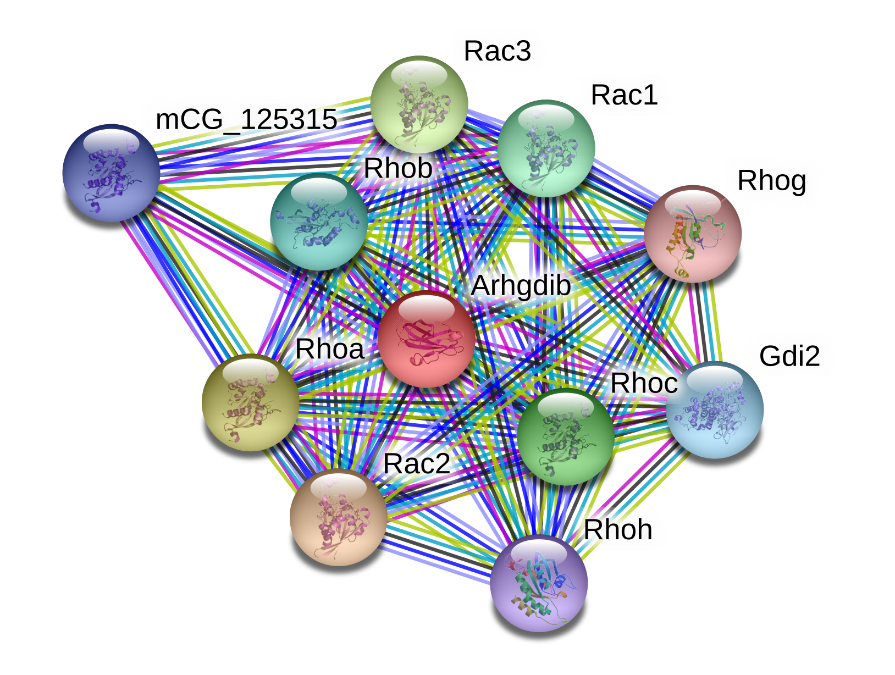


**Supplementary figure 5.** STRING enriched analysis of Arhgdib protein interactions in the Agarikon.1 and 5-fluorouracil down-accumulated group.


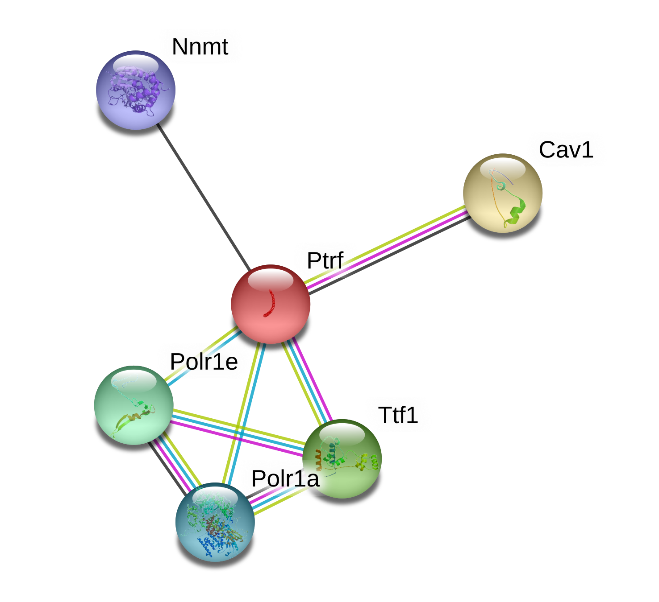


**Supplementary figure 6.** STRING enriched analysis of Ptrf (Cavin-1) protein interactions in the Agarikon.1 and 5-fluorouracil down-accumulated group.


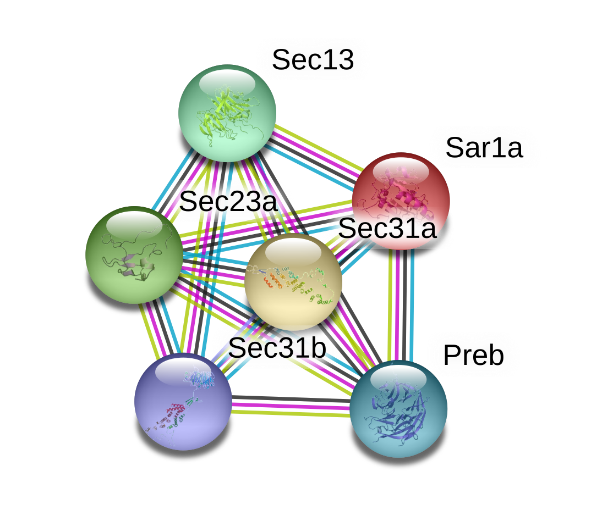


**Supplementary figure 7.** STRING enriched analysis of Sar1a protein interactions in the Agarikon.1 and 5-fluorouracil down-accumulated group.


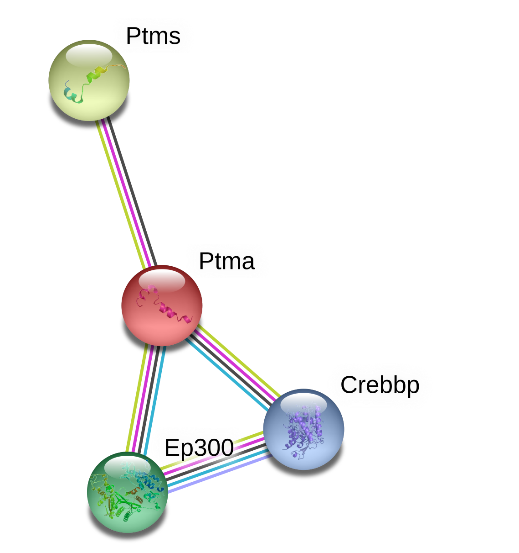


**Supplementary figure 8.** STRING enriched analysis of Ptma protein interactions in the 5-fluorouracil up-accumulated group.


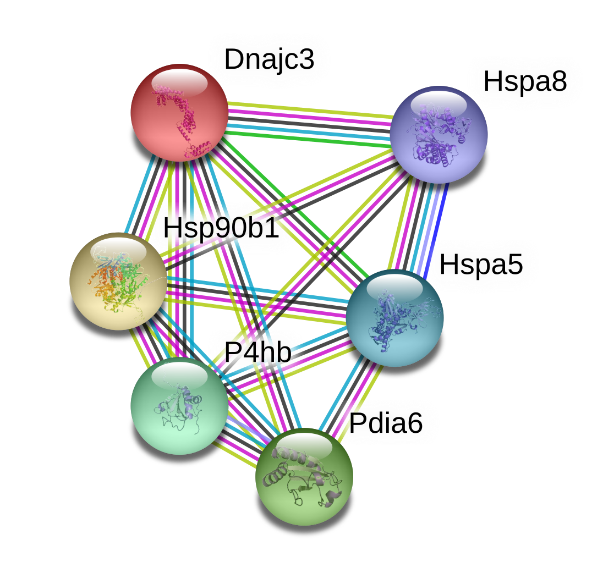


**Supplementary figure 9.** STRING enriched analysis of Dnajc3 protein interactions in the Agarikon.1, 5-fluorouracil and Agarikon.1 + 5-fluorouracil up-accumulated groups.

**Legend for Supplementary figures 2-9:**


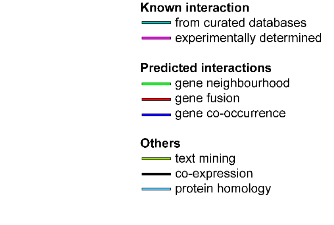

Supplement: Supplementary file 10 [file DataSheet_10.docx]
